# Effects of variable viscosity, concentration and heat variation on MHD oscillatory flow for Bingham fluid through an inclined porous channel

**DOI:** 10.12688/f1000research.172909.2

**Published:** 2026-06-23

**Authors:** Dheia G. Salih Al-Khafajy, Amal Al-Hanaya, Munirah Aali Alotaibi

**Affiliations:** 1 Department of Mathematics, University of Al-Qadisiyah, Diwaniya, Iraq; 2 Mathematical Sciences, College of Science, Princess Nourah bint Abdulrahman University, Riyadh, Saudi Arabia

**Keywords:** Bingham fluid, thermal transfer, concentration, magnetohydrodynamic (MHD), inclined porous channel

## Abstract

**Background:**

Magnetohydrodynamic oscillatory flows of non-Newtonian fluids in porous channels arise in many industrial and geophysical applications. Understanding the combined influence of variable viscosity, heat generation, and concentration is essential for accurate flow prediction.

**Methods:**

A mathematical model for unsteady MHD oscillatory flow of a Bingham fluid through an inclined porous channel was formulated. The governing nonlinear partial differential equations for momentum, energy, and concentration were nondimensionalized and solved using the separation of variables technique. Numerical evaluation and graphical analysis were performed using Wolfram Mathematica.

**Results:**

The results show that increasing heat generation and radiation parameters enhances fluid temperature and velocity, while higher magnetic and oscillation parameters suppress flow motion. Concentration was found to increase with higher oscillation frequency and Péclet number, whereas Schmidt and chemical reaction parameters reduced mass diffusion. Variable viscosity significantly amplified velocity compared to constant-viscosity cases.

**Conclusions:**

The study demonstrates that temperature-dependent viscosity and yield-stress effects strongly control MHD oscillatory Bingham fluid flow in inclined porous channels. The results are relevant to engineering systems involving non-Newtonian transport with thermal and mass diffusion effects. The novelty of the present formulation lies in combining Bingham yield-stress behavior, temperature-dependent viscosity, porous-medium resistance, magnetic-field effects, heat generation, radiation, and concentration transport within a single analytical framework.

## 1. Introduction

A Bingham fluid is a category of non-Newtonian fluid that exhibits solid-like behavior at low-stress levels and flows as a viscous liquid under elevated stress levels. They demonstrate yield stress, indicating that the fluid remains static until the applied stress is above a specific critical threshold. Below this yield stress, the material exhibits rigid body behavior. Upon surpassing the yield stress, the flow behavior becomes linear, characterized by a constant viscosity. The linear correlation between stress and strain rate manifests similarly to that observed in Newtonian fluids. Typical instances encompass toothpaste, mayonnaise, and concrete. These materials necessitate a certain force to initiate flow. Bingham fluids are significant in numerous industrial and scientific applications, especially where materials necessitate exact regulation of flow characteristics. Bird et al. ^1^ examined the behaviour of a Bingham fluid within a hard circular tube. Kapur ^2^ introduced several mathematical models, such as Cais-son, the Bingham, and Herschel-Bulkley models. Rathy ^3^ examined the fluid dynamics of a Bingham fluid within a channel and an annulus featuring impermeable barriers. Vajravelu et al. ^4^ conducted an experimented on the flow of Bingham fluid in an annular tube with a porous wall. Ramakrishna et al ^5^ investigated the flow behaviour of a Bingham fluid on a permeable bed in an enclosed channel. Goverdhan ^6^ examined the flow of Bingham fluid in a porous channel. Narahari et al. ^7^ Investigate the movement of a Bingham fluid between two porous substrates, focusing on its unsteady behaviour. Recently, Murthy et al. ^8^ focused on analysing the flow of a Bingham fluid, which is not stable, in contact with a Newtonian fluid within two parallel plates. The objective is to determine the velocity field, mass flow rates, and interface velocity. Tsangaris et al. ^9^ conducted on the movement of a Bingham fluid between two porous walls that are parallel to each other. One wall moves steadily in the same direction as the other wall, which remains stationary. At the same time, there is a pressure difference throughout the length of the wall, and there is a flow of fluid across the walls due to their porous nature. Adnan and Abdulhadi ^10^ performed an investigation on the influence of an inclined magnetic field on the flow of incompressible Bingham plastic fluid within an inclined symmetric channel. The study also considered the effects of mass transfer and heat transfer. Slip circumstances are utilised for heat transmission and focus. Lakshminarayana et al. ^11^ examined the impact of wall slip circumstances, elasticity wall characteristics, and heat transfer on the movement of conducting Bingham fluid in an irregular channel using the presumptions of a lengthy wavelength and low Reynolds number. The current research by Mahabaleshwar et al. ^12^ investigates how radiation and chemical reactions affect the two-dimensional boundary layer flow of a bi-viscous Bingham fluid on a thermosolutal Marangoni boundary that is accompanied by a magnetic field and thermal source or sink. A mathematical model is developed using the Navier-Stokes equations to represent the physical flow problem. In order to convert these nonlinear PDEs into a system of nonlinear ODEs, they employed a similarity transformation. The study conducted by Basavarajappa et al. ^13^ examines the multilayer flow of a bi-viscous Bingham fluid within a vertical slab with a hybrid nanofluid, using the nonlinear Boussinesq approximation. Recent studies have continued to examine oscillatory and unsteady MHD convection in thermally active media. Afridi et al. ^14^ analyzed entropy generation minimization and heat-transfer enhancement in MHD oscillatory ferrofluid flow, emphasizing the role of magnetic control in thermal transport. Ullah et al. ^15^ studied oscillatory mixed convection around a horizontal circular cylinder embedded in a porous medium while considering temperature-dependent density effects. Nandkeolyar et al. ^16^ investigated unsteady hydromagnetic natural convection of a dusty fluid past a moving vertical plate with ramped temperature and thermal radiation. These studies show that unsteadiness, magnetic effects, buoyancy, and porous-medium resistance remain important mechanisms in convective transport problems.

Heat transport in Bingham fluids entails intricate interplay between the fluid’s rheological features and thermal aspects. The existence of a yield stress, which regulates flow commencement, profoundly influences heat transfer processes. Vradis et al. ^17^ numerically resolved the concurrent evolution of thermal fields and hydrodynamic at the entry region of a cylindrical pipe for a non-Newtonian Bingham-type fluid by employing the completely elliptic mathematical models of continuity, momentum, and energy. Mustafa et al. ^18^ conducted a heat transfer simulation for the swirling flow of a Bingham fluid constrained by a permeable rotation disk. The influence of concentration and temperature variations on magnetohydrodynamic (MHD) oscillatory flow in a porous media has numerous practical implications in the fields of engineering, industry, medical research, and issues related to the extraction and transportation of petroleum. Hamza et al. ^19^ conducted a study on the impact of slip condition, radiative heat transfer, and transverse magnetic field on the unsteady flow of a conducting optical thin fluid via a channel equipped with a porous media. Khudair and Al-Khafajy ^20^ proposed a heat transfer model for MHD oscillating flow of Williamson fluid over a porous plate, considering two different forms of flow. Al-Aridhee and Al-Khafajy ^21^ examined the impact of mass and heat transfer on the peristaltic movement of MHD flow of a non-Newtonian Jeffrey fluid via a cylindrical porous media channel. The investigation focuses on the flow within a wave frame of reference that is travelling at the velocity of the wave. Al-Khafajy and Labban ^22^ conducted a study on the combined impact of concentration and thermo-diffusion on the fluctuating flow of an incompressible Carreau fluid via an angled permeable channel. Liu et al. ^23^ investigate the steady flow and thermal transfer of Bingham fluid over a spinning disk of limited radius with radially varying thickness in the boundary layer. Eldabe et al. ^24^ examined the flow of non-Newtonian Bingham blood fluid down an irregular conduit. The fluid exhibits electrical conductivity, and an external uniform magnetic field is added to this motion. Heat and mass transmission are considered, leading to an examination of the Dufour and Soret effects. Al-Khafajy and Mohammed ^25^ examined a mathematical model elucidating the effects of thermal transfer on the oscillating flow of Bingham fluid with changing viscosity in a porous channel within the context of magnetohydrodynamics. Salahuddin et al. ^26^ performed a study analyzing numerical behavior utilizing the Adams-Bashforth predictor-corrector method of numerical analysis for the Williamson fluid model, considering variable viscosity, natural convection, and an angled magnetic field. Additionally, thermal radiation, Joule heating, and heat source/sink effects are incorporated into the thermal considerations. Akram et al. ^27^ studied the peristaltic flow of Bingham fluids under an inclined magnetic field, while Humnekar and Darbhasayanam ^28^ investigated variable-viscosity nanofluid flow in inclined porous media. These recent studies highlight the ongoing need for improved formulations that incorporate thermal, magnetic, and viscous variations simultaneously. Recent works have also extended non-Newtonian flow analysis by incorporating advanced thermal and concentration mechanisms. Panda et al. ^29^ examined Marangoni convection in a tangent hyperbolic fluid with Cattaneo–Christov heat and mass flux, buoyancy, and activation energy effects. Sneha et al. ^30^ considered MHD Marangoni nano-boundary-layer flow with carbon nanotubes and thermal radiation, highlighting the influence of magnetic and radiative effects on heat transfer. More closely related to yield-stress rheology, Sachhin et al. ^31^ analyzed thermodynamic and viscosity-ratio effects in a non-Newtonian tetra-Bingham nanofluid flow, showing the importance of rheological parameters in controlling transport behavior. 

In an inclined channel, the influence of gravity is crucial in propelling the flow. The gravitational force acting along the slope of the channel affects the overcoming of yield stress, allowing the fluid to flow. In the case of Bingham fluids, flow takes place when the shear stress surpasses the yield stress. The angle of inclination in a channel influences the critical shear stress required to initiate flow. Lakshminarayana et al. ^32^ investigated the concurrent impacts of heated Joules and slip on the peristaltic flow of a Bingham fluid within an inclination tapered permeable channel featuring elastic walls. Mohammed and Al-Khafajy ^33^ examined the impact of temperature and concentration on magnetohydrodynamic oscillatory flow of Bingham fluid with varying viscosity in a sloped channel. umbinarasaiah ^34^ conducted a numerical investigation of entropy generation in an incompressible Casson fluid moving through an inclined permeable channel subjected to magnetic influence. Jha and Aina ^35^ offered a mathematical model for the complete magnetohydrodynamic mixed convection flow of an electrically conducting, viscous, incompressible fluid in an inclined permeable channel subject to time-periodic boundary constraints.

Although the above studies provide useful insights into oscillatory MHD convection, porous-medium transport, non-Newtonian rheology, and coupled heat and mass transfer, most of them address these mechanisms separately or in different geometrical configurations. Analytical studies combining oscillatory MHD mixed convection, Bingham yield-stress behavior, variable viscosity, porous-medium resistance, inclination effects, and coupled thermal and concentration transport in a single channel-flow formulation remain limited. Therefore, the novelty of the present work lies in developing an analytical model that brings these interacting mechanisms together and clarifies how the competition between buoyancy forces, Lorentz-force damping, yield-stress resistance, viscosity variation, and porous drag controls the velocity, temperature, and concentration fields.

This study comprises five sections. The initial section is the introduction, which encompasses a historical review of the factors being examined. The second involves constructing the mathematical model. The third portion presents the resolution to the issue. The fourth portion encompasses an analysis of the outcomes via the function diagrams we acquired. The investigation was ended with a discussion and conclusions.

## 2. Mathematical formulation

Let us consider the flow of a non-Newtonian (Bingham) fluid with variable viscosity under the effects of radioactive heat transfer and electrically-applied magnetic field as depicted through an inclined porous channel with a width of h ( Figure 1). Fluids are supposed to have minimal electromagnetic power produced with a low electrical conductivity. We think of the system of Cartesian coordinates so that is the velocity vector.

**Figure 1.**
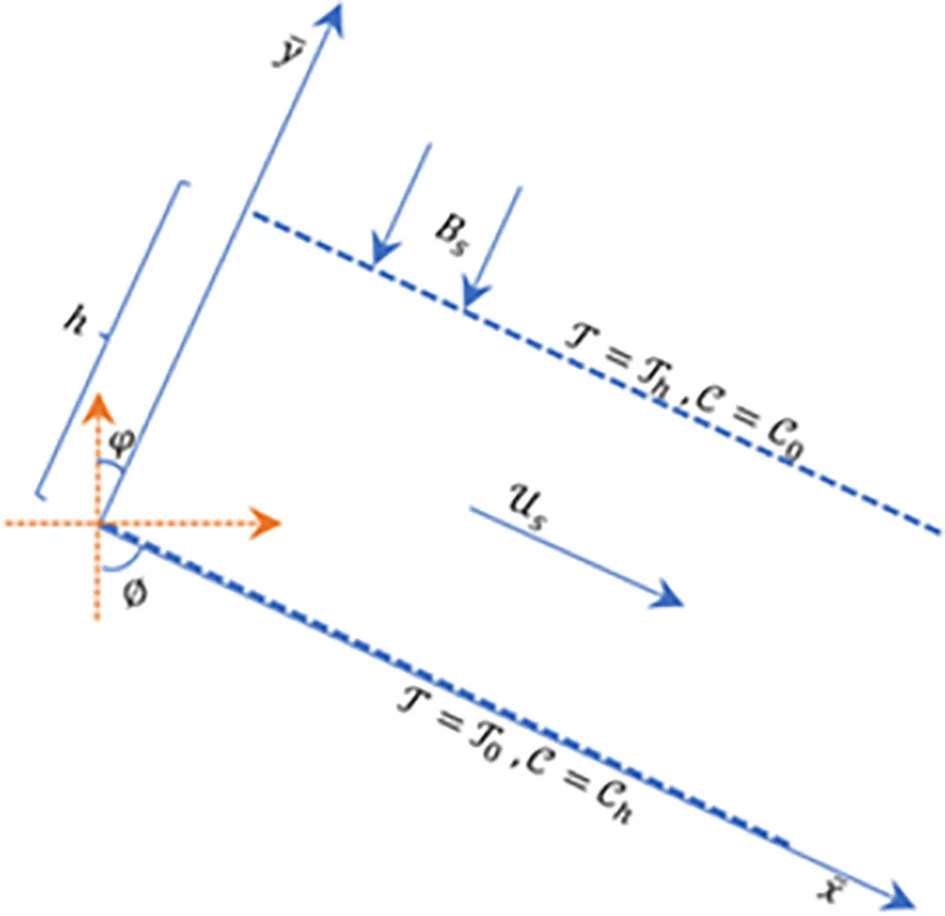
Physical model of the inclined porous channel.

The basic equations governing the problem are provided as:

The continuity equation is given by:

∇.U=0 (1)

The momentum equations:

$$\rho \left(\bar{U}.\nabla \right)\bar{U}=\nabla .\bar{S}+\bar{J}\times \bar{B}-\frac{\mu \left(\bar{T}\right)}{k}\bar{U}+\mathrm{\rho g}B_{T}\sin\varphi ∆\bar{T}+\mathrm{\rho g}B_{C}\sin\varphi ∆\bar{C}+\mathrm{\rho g}\left(\text{isin}\left(\phi \right)-\text{jcos}\left(\phi \right)\right)$$ (2)

The concentration equation:

$$\left(\bar{U}.\nabla \right)\bar{C}=\nabla .\left(\frac{D_{C}T_{d}}{T_{m}}\nabla \bar{T}+D_{C}\nabla \bar{C}\right)-K_{r}^{*}∆\bar{C}$$ (3)

The temperature equation:

$$\rho c_{T}\left(\bar{U}.\nabla \right)\bar{T}=\nabla .\left(K_{T}\nabla \bar{T}\right)+∆.Q_{T}+Q_{H}∆\bar{T}$$ (4)

where $\bar{U}=\left(\bar{u}\left(\bar{y},\bar{t}\right),0,0\right)$ is the velocity field, $\bar{T}=\bar{T}\left(\bar{y},\bar{t}\right)$ “temperature”, $\bar{C}=\bar{C}\left(\bar{y},\bar{t}\right)$ “concentration”, $\bar{J}\times \bar{B}=-\sigma B_{s}^{2}\sin^{2}\varphi \;ui$ “Lorentz force for inclined magnetic field strength”, ^33^ σ is a conductivity of the fluid, k is a permeability, ρ “fluid density”, g “gravity field”, $c_{T}$ “specific heat at constant pressure”, $K_{T}$ “thermal conductivity”, $Q_{H}$ “heat generation”, $D_{C}$ “coefficient of mass diffusivity” and $T_{d}$ “thermal diffusion ratio”, $∆.Q_{T}=4\alpha^{2}\left(\bar{T}-T_{0}\right)$ ”radiation heat flux”, ^36^ α “radiation absorption”, (0≤ϕ≤π) is the angle between the centre channel and the ground acceleration. The corresponding boundary conditions are given below:

$$\bar{u}=0,\bar{T}=T_{0},\bar{C}=C_{h}\;\mathrm{at}\;\bar{y}=0\;\text{and}\;\bar{u}=0,\bar{T}=T_{h},\bar{C}=C_{0}\;\mathrm{at}\;\bar{y}=h$$ (5)

The basic equation for the Bingham fluid, ^32^ given by:

$$\bar{S}=-\bar{p}I+\bar{s}\;\text{and}\;\bar{s}=\left\{ \begin{array}{ccc} \left(\mu \left(\bar{T}\right)+\frac{\tau_{0}}{\left(\frac{\partial \bar{u}}{\partial \bar{y}}\right)}\right)\left(\nabla \bar{U}+{\left(\nabla \bar{U}\right)}^{T}\right) & \text{for} & \tau \geq \tau_{0}\\ 0 & \text{for} & \tau <\tau_{0} \end{array}\right.$$ (6)

Where $\bar{p}$ “pressure”, I “unit tensor”, $\mu_{c}$ “fluid viscosity”, $\tau_{0}$ “yield stress”, and $\bar{\dot{\gamma }}$ “shear rate”.

When compensating for the velocity vector and the concentration and temperature functions, taking into account the magnetic field generated by the passage of a simple electric current over a porous wall of the inclined flow channel, and by compensating for the shear stress ( Equation 6), and after simplifying, we rewrite the nonlinear partial differential system (1)-(4) as follows:

$$\frac{\partial \bar{u}}{\partial \bar{x}}+\frac{\partial \bar{v}}{\partial \bar{y}}=0$$ (7)

$$\rho \left(\frac{\partial \bar{u}}{\partial \bar{t}}+\bar{u}\frac{\partial \bar{u}}{\partial \bar{x}}+\bar{v}\frac{\partial \bar{u}}{\partial \bar{y}}\right)=-\frac{\partial \bar{p}}{\partial \bar{x}}+\frac{\partial {\bar{s}}_{11}}{\partial \bar{x}}+\frac{\partial {\bar{s}}_{12}}{\partial \bar{y}}-\sigma B_{s}^{2}\sin^{2}\varphi \;\bar{u}-\frac{\mu \left(\bar{T}\right)}{k}\bar{u}+\mathrm{\rho g}B_{T}\sin\varphi \left(\bar{T}-T_{0}\right)+\mathrm{\rho g}B_{C}\sin\varphi \left(\bar{C}-C_{0}\right)+\mathrm{\rho g}\sin\phi$$ (8)

$$\rho \left(\frac{\partial \bar{v}}{\partial \bar{t}}+\bar{u}\frac{\partial \bar{v}}{\partial \bar{x}}+\bar{v}\frac{\partial \bar{v}}{\partial \bar{y}}\right)=-\frac{\partial \bar{p}}{\partial \bar{y}}+\frac{\partial {\bar{s}}_{21}}{\partial \bar{x}}+\frac{\partial {\bar{s}}_{22}}{\partial \bar{y}}-\frac{\mu \left(\bar{T}\right)}{k}\bar{v}$$ (9)

$$\frac{\partial \bar{C}}{\partial \bar{t}}+\bar{u}\frac{\partial \bar{C}}{\partial \bar{x}}+\bar{v}\frac{\partial \bar{C}}{\partial \bar{y}}=\frac{D_{C}T_{d}}{T_{m}}\left(\frac{\partial^{2}\bar{T}}{\partial {\bar{x}}^{2}}+\frac{\partial^{2}\bar{T}}{\partial {\bar{y}}^{2}}\right)+D_{C}\left(\frac{\partial^{2}\bar{C}}{\partial {\bar{x}}^{2}}+\frac{\partial^{2}\bar{C}}{\partial {\bar{y}}^{2}}\right)-K_{r}^{*}\left(\bar{C}-C_{0}\right)$$ (10)

$$c_{T}\rho \left(\frac{\partial \bar{T}}{\partial \bar{t}}+\bar{u}\frac{\partial \bar{T}}{\partial \bar{x}}+\bar{v}\frac{\partial \bar{T}}{\partial \bar{y}}\right)=K_{T}\left(\frac{\partial^{2}\bar{T}}{\partial {\bar{x}}^{2}}+\frac{\partial^{2}\bar{T}}{\partial {\bar{y}}^{2}}\right)-4\alpha^{2}\left(T_{0}-\bar{T}\right)+Q_{H}\left(\bar{T}-T_{0}\right)$$ (11)

The stress components are given by:

$${\bar{s}}_{11}={\bar{s}}_{22}=0\;\text{and}\;{\bar{s}}_{12}={\bar{s}}_{21}=\left(\mu \left(\bar{T}\right)+\frac{\tau_{0}}{\left(\frac{\partial \bar{u}}{\partial \bar{y}}\right)}\right)\frac{\partial \bar{u}}{\partial \bar{y}}=\mu \left(\bar{T}\right)\frac{\partial \bar{u}}{\partial \bar{y}}+\tau_{0}$$ (12)

By substituting the equation (12) into the governing equations and after simplifying, we obtain:

$$\rho \frac{\partial \bar{u}}{\partial \bar{t}}=-\frac{\partial \bar{p}}{\partial \bar{x}}+\frac{\partial }{\partial \bar{y}}(\mu \left(\bar{T}\right)\frac{\partial \bar{u}}{\partial \bar{y}}+\tau_{0})-\sigma B_{s}^{2}\sin^{2}\varphi \;\bar{u}-\frac{\mu \left(\bar{T}\right)}{k}\bar{u}+\mathrm{\rho g}\sin\varphi \left(B_{T}\left(\bar{T}-T_{0}\right)+B_{C}\left(\bar{C}-C_{0}\right)\right)+\mathrm{\rho g}\sin\phi$$ (13)

$$\frac{\partial \bar{p}}{\partial \bar{y}}=0$$ (14)

$$\frac{\partial \bar{C}}{\partial \bar{t}}=\frac{D_{C}T_{d}}{T_{m}}\frac{\partial^{2}\bar{T}}{\partial {\bar{y}}^{2}}+D_{C}\frac{\partial^{2}\bar{C}}{\partial {\bar{y}}^{2}}-K_{r}^{*}\left(\bar{C}-C_{0}\right)$$ (15)

$$c_{T}\rho \frac{\partial \bar{T}}{\partial \bar{t}}=K_{T}\frac{\partial^{2}\bar{T}}{\partial {\bar{y}}^{2}}+\left(4\alpha^{2}+Q_{H}\right)\left(\bar{T}-T_{0}\right)$$ (16)

## 3. Method of solution

The equation (14) shows that the pressure does not depend on y . To solve the above system of equations, we use its non-dimensional conditions as follows:

By substituting equations from Table 1 into equations (13), (15), (16) and the equations of boundary conditions equation (5), we have the following non-dimensional equations:

$$R_{e}\frac{\partial u}{\partial t}=-\frac{\partial p}{\partial x}+\mu \left(T\right)\frac{\partial^{2}u}{\;\partial y^{2}}+\frac{\partial u}{\partial y}\frac{\partial \mu \left(T\right)}{\partial y}-\left(M^{2}\sin^{2}\varphi +\frac{\mu \left(T\right)}{\mathrm{Da}}\right)u+\sin\varphi \left(G_{T}T+G_{C}C\right)+\frac{R_{e}}{F_{r}}\sin\phi$$ (17)

$$\frac{\partial C}{\partial t}=S_{T}\frac{\partial^{2}T}{\;\partial y^{2}}+\frac{1}{S_{C}}\frac{\partial^{2}C}{\;\partial y^{2}}-K_{C}C$$ (18)

$$\mathrm{Pe}\frac{\partial T}{\partial t}=\frac{\partial^{2}T}{\;\partial y^{2}}+\left(K_{T}+Q_{H}\right)T$$ (19)

u=0,T=0,C=1aty=0andu=0,T=1,C=0aty=1 (20)

**Table 1.** Dimensionless parameters and their physical significance.

Symbol	Parameter name	Definition/Expression	Physical interpretation
Re	Reynolds number	Re=ρhUsμc	Ratio of inertial to viscous forces; characterizes flow regime.
Pe	Péclet number	$\mathrm{Pe}=\frac{\rho hU_{s}c_{T}}{K_{T}}$	Measures the relative importance of convective to conductive heat transfer.
Sc	Schmidt number	$\mathrm{Sc}=\frac{\mu_{c}}{{\mathrm{\rho D}}_{C}}$	Ratio of momentum diffusivity to mass diffusivity; governs concentration boundary layer thickness.
M	Magnetic parameter (Hartmann number squared)	$M=\sqrt{\frac{\sigma B_{s}^{2}h^{2}}{\mu_{c}}}$	Represents the relative influence of Lorentz (magnetic) forces over viscous forces.
Da	Darcy number	$\mathrm{Da}=\frac{k}{h^{2}}$	Indicates the permeability effect of the porous medium on the flow.
$F_{r}$	Froude number	$F_{r}=\frac{U_{s}^{2}}{\mathrm{gh}}$	Expresses the ratio of inertial forces to gravitational forces.
$B_{n}$	Bingham number	$B_{n}=\tau_{0}\frac{h}{\mu_{c}U_{s}}$	Quantifies yield stress effects; higher values indicate stronger resistance before flow begins.
$G_{T}$	Thermal Grashof number	$G_{T}=\frac{\mathrm{\rho g}B_{T}h^{2}\left(T_{h}-T_{0}\right)}{\mu_{c}U_{s}}$	Represents buoyancy forces due to temperature differences.
$G_{C}$	Solutal Grashof number	$G_{C}=\frac{\mathrm{\rho g}B_{C}h^{2}\left(C_{h}-C_{0}\right)}{\mu_{c}U_{s}}$	Represents buoyancy effects due to concentration differences.
$K_{T}$	Radiation parameter	$K_{T}=\frac{4\alpha^{2}h^{2}}{K_{T}}$	Measures the contribution of radiative heat transfer relative to conduction.
$Q_{H}$	Heat generation parameter	$Q_{H}=\frac{Q_{H}h^{2}}{K_{T}}$	Quantifies internal heat generation or absorption within the fluid.
$S_{T}$	Soret number	$S_{T}=\frac{D_{C}T_{d}\left(T_{h}-T_{0}\right)}{hT_{m}U_{s}\left(C_{h}-C_{0}\right)}$	Captures thermodiffusion effects (mass flux due to temperature gradients).
$K_{C}$	Chemical reaction parameter	$K_{C}=\frac{hK_{r}^{*}}{U_{s}}$	Represents the rate of chemical reaction relative to convective transport.
ϕ	Channel inclination angle	-	Angle between channel axis and horizontal plane; controls gravity component along the flow.
φ	Magnetic field inclination angle	-	Angle between magnetic field and vertical direction; controls Lorentz force orientation.

### 3.1 Solution of the problem

This section includes solving a system of differential equations. We begin by solving the heat equation, passing through the concentration equation, and then we end by solving the velocity equation.

**3.1.1 Solution of the heat and concentration equations**

Using the separating variables method to solve the heat equation (19) and the concentration equation (18) with the boundary conditions equation (20), respectively. Let ω be the frequency of oscillation, and let

$$T\left(y,t\right)=T_{0}\left(y\right)\;e^{\mathrm{i\omega t}}\;\text{and}\;C\left(y,t\right)=C_{0}\left(y\right)e^{\mathrm{i\omega t}}$$ (21)

By substituting equation (21) into equations (19) and (18), respectively, and after simplifying the two equations, we obtain

$$\frac{\partial^{2}C_{0}\left(y\right)}{\;\partial y^{2}}+S_{C}S_{T}\frac{\partial^{2}T_{0}\left(y\right)}{\;\partial y^{2}}-\left(S_{C}K_{C}+\mathrm{i\omega}S_{C}\right)\;C_{0}\left(y\right)=0$$

$$\frac{\partial^{2}T_{0}\left(y\right)}{\;\partial y^{2}}+\left(K_{T}+Q_{H}-\text{Peiω}\right)T_{0}\left(y\right)=0$$

with boundary conditions $T_{0}\left(0\right)=C_{0}\left(1\right)=0$ and $T_{0}\left(1\right)=C_{0}\left(0\right)=e^{-\mathrm{i\omega t}}$ .

The solution of temperature equation is

$$T\left(y,t\right)=\mathrm{Csc}\left[\sqrt{H}\right]\mathrm{Sin}\left[\sqrt{H}y\right]$$

The solution of concentration equation is

$$C\left(y,t\right)={\mathrm{e}}^{\mathrm{it\omega}}\left(\left(-\frac{{\mathrm{e}}^{-\mathrm{it\omega}+\sqrt{\mathrm{i\omega}+K_{C}}\sqrt{S_{C}}}-\frac{{\mathrm{e}}^{-\mathrm{it\omega}}HS_{C}S_{T}}{H+\mathrm{i\omega}S_{C}+K_{C}S_{C}}}{{\mathrm{e}}^{-\sqrt{\mathrm{i\omega}+K_{C}}\sqrt{S_{C}}}-{\mathrm{e}}^{\sqrt{\mathrm{i\omega}+K_{C}}\sqrt{S_{C}}}}\right){\mathrm{e}}^{-y\sqrt{\mathrm{i\omega}+K_{C}}\sqrt{S_{C}}}+\left(\frac{{\mathrm{e}}^{-\mathrm{it\omega}}\left(-H-\mathrm{i\omega}S_{C}-K_{C}S_{C}+{\mathrm{e}}^{\sqrt{\mathrm{i\omega}+K_{C}}\sqrt{S_{C}}}HS_{C}S_{T}\right)}{\left(-1+{\mathrm{e}}^{2\sqrt{\mathrm{i\omega}+K_{C}}\sqrt{S_{C}}}\right)\left(H+\mathrm{i\omega}S_{C}+K_{C}S_{C}\right)}\right){\mathrm{e}}^{y\sqrt{\mathrm{i\omega}+K_{C}}\sqrt{S_{C}}}-\frac{{\mathrm{e}}^{-\mathrm{it\omega}}H\mathrm{Csc}\left[\sqrt{H}\right]\mathrm{Sin}\left[\sqrt{H}y\right]S_{C}S_{T}}{H+\mathrm{i\omega}S_{C}+K_{C}S_{C}}\right)$$

where H $=K_{T}+Q_{H}-\text{Peiω}$ .

**3.1.2 Solution of the momentum equations**

The Reynolds model for the variation of viscosity with temperature is μ(T)=exp(−ϵT) , taking the Maclaurin’s expansion, we get μ(T)=1−ϵT , ϵ≪1 .

Using the separating variables method to solve the momentum equation (17) with the boundary conditions equation (20). Let

$$u\left(y,t\right)=u_{0}\left(y\right)e^{\mathrm{i\omega t}}\;\text{and}\;\frac{\mathrm{dp}}{\mathrm{dx}}=-\lambda e^{\mathrm{i\omega t}}$$ (22)

Where λ is a real pressure constant. Substituting Maclaurin’s formula for the fluid viscosity variable in addition to the (22) equation in the (17) equation, we get

$$R_{e}\mathrm{i\omega}\;u_{0}\left(y\right)=\lambda +\left(1-\epsilon T\right)\frac{\partial^{2}\left(u_{0}\left(y\right)\right)}{\;\partial y^{2}}-\epsilon \frac{\partial \left(u_{0}\left(y\right)\right)}{\partial y}\frac{\partial T}{\partial y}-\left(M^{2}\sin^{2}\varphi +\frac{1}{\mathrm{Da}}\right)u_{0}\left(y\right)+\frac{\epsilon T}{\mathrm{Da}}u_{0}\left(y\right)+\sin\varphi \left(G_{T}T_{0}+G_{C}C_{0}\right)+e^{-\mathrm{i\omega t}}\frac{R_{e}}{F_{r}}\sin\phi$$ (23)

The equation’s solution will be examined in two distinct cases.

**Case I (**when ϵ=0 )

In the specific instance when ϵ=0 , indicating that the viscosity remains constant, we obtain from (23)

$$\frac{\partial^{2}u_{0}\left(y\right)}{\;\partial y^{2}}-\left(M^{2}\sin^{2}\varphi +\frac{1}{\mathrm{Da}}+\mathrm{i\omega}R_{e}\;\right)u_{0}\left(y\right)=-\left(\lambda +e^{-\mathrm{i\omega t}}\left(G_{T}\sin\varphi T+G_{C}\sin\varphi C+\frac{R_{e}}{F_{r}}\sin\phi \right)\right)$$ (24)

with boundary conditions: $u_{0}\left(0\right)=u_{0}\left(1\right)=0.$

Due to the complexity of solving the velocity equation, we shall analyze the behavior of the solution by graphing the function rather than deriving its formula.

**Case II (**when ϵ≠0)

Equation (23) is a nonlinear differential equation and it is hard to find an exact solution, so we will use the perturbation technique to find the solution to the problem as follows:

$$u_{0}\left(y\right)=u_{00}\left(y\right)+\epsilon u_{01}\left(y\right)+o\left(\epsilon^{2}\right)$$ (25)

Substituting equation (25) in equation (23), we obtain:

$$R_{e}\mathrm{i\omega}\;\left(u_{00}\left(y\right)+\epsilon u_{01}\left(y\right)\right)=\lambda +\left(1-\epsilon T\right)\frac{\partial^{2}}{\;\partial y^{2}}\left(u_{00}\left(y\right)+\epsilon u_{01}\left(y\right)\right)-\epsilon \frac{\partial T}{\partial y}\frac{\partial }{\partial y}\left(u_{00}\left(y\right)+\epsilon u_{01}\left(y\right)\right)-\left(M^{2}\sin^{2}\varphi +\frac{1}{\mathrm{Da}}\right)\left(u_{00}\left(y\right)+\epsilon u_{01}\left(y\right)\right)+\frac{\epsilon T}{\mathrm{Da}}\left(u_{00}\left(y\right)+\epsilon u_{01}\left(y\right)\right)+\sin\varphi G_{T}T_{0}+\sin\varphi G_{C}C_{0}+e^{-\mathrm{i\omega t}}\frac{R_{e}}{F_{r}}\sin\phi$$

Equating the like powers of ϵ , we obtain the following results presented in the forthcoming subsections:

i. **Zero-order system**

$$\frac{\partial^{2}u_{00}\left(y\right)}{\;\partial y^{2}}-\left(M^{2}\sin^{2}\varphi +\frac{1}{\mathrm{Da}}+R_{e}\mathrm{i\omega}\;\right)u_{00}\left(y\right)=-\left(\lambda +G_{T}\sin\varphi T_{0}+\sin\varphi G_{C}C_{0}+e^{-\mathrm{i\omega t}}\frac{R_{e}}{F_{r}}\sin\phi \right)$$ (26)

It is consistent with the Case I.

ii. **First-order system**

$$\frac{\partial^{2}u_{01}\left(y\right)}{\;\partial y^{2}}-\left(M^{2}\sin^{2}\varphi +\frac{1}{\mathrm{Da}}+R_{e}\mathrm{i\omega}\;\right)u_{01}\left(y\right)=T\left(\frac{\partial^{2}u_{00}\left(y\right)}{\;\partial y^{2}}-\frac{1}{\mathrm{Da}}u_{00}\left(y\right)\right)+\frac{\partial T}{\partial y}\frac{\partial u_{00}\left(y\right)}{\partial y}$$ (27)

with the boundary conditions: $u_{01}\left(0\right)=u_{01}\left(1\right)=0$

The nonlinear characteristics of equation (27) render the derivation of an exact analytical formula for velocity difficult. Consequently, we employed an approximate method utilizing perturbation and separation of variables to examine the effects of varying viscosity and other flow characteristics. In the current investigation, the governing equations initially excluded the explicit description of the yield-stress factor related to Bingham rheology. To overcome this constraint and offer a more physically accurate depiction of the flow, the formulation has been enhanced to explicitly include the yield criterion and the Bingham number (B), which denotes the ratio of yield to viscous stresses. The updated formulation is detailed in the subsequent subsection.

### 3.2 Bingham regularization and yield surface

The momentum equation was revised to explicitly include the yield-stress impact through the Papanastasiou regularization [Papanastasiou, 1987]. This formulation eliminates discontinuities between yielded and unyielded regions, facilitating seamless numerical analysis. The effective shear stress is written as:

$$\tau =\tau_{ο}\;\left[1-\exp\left(-m\dot{\gamma }\right)\right]+\mu c\;\dot{\gamma },$$

where τ _0_ represents the yield stress. Shear rate is denoted as $\dot{\gamma }\;$ , and *m* represents a significant regularization parameter that governs the abruptness of the transition between the unyielded and yielded states. As *m* approaches infinity, this statement simplifies to the traditional Bingham model.

The **yield surface** $y=y_{p}$ is defined as the location where the local shear stress equals the yield stress, i.e., $|\tau \left(y_{p}\right)|=\tau_{0}$ . For $|\tau |<\tau_{0}$ , the material behaves as a rigid plug region, while for $|\tau |>\tau_{0}$ , it flows as a viscous fluid.

This regularized form was substituted into the non-dimensional momentum equation (17), allowing explicit inclusion of the **Bingham number** $B_{n}=\tau_{0}h/\left(\mu_{c}U_{0}\right)$ . The modified governing equation therefore becomes:

Re∂u∂t=−∂p∂x+μ(T)∂2u∂y2+∂μ(T)∂y∂u∂y−(M2sin2ϕ+μ(T)Da)u+Bn∂u∂y+sinϕ(GTT+GCC)+ReFrsinφ.

This expression correctly accounts for yield-stress effects and ensures the influence of both yielded and unyielded regions is captured in the flow field. The velocity profiles in Figures 8– 24 were reinterpreted under this framework, showing clear flattening within the central plug region, consistent with expected Bingham behaviour.

**Figure 2.**
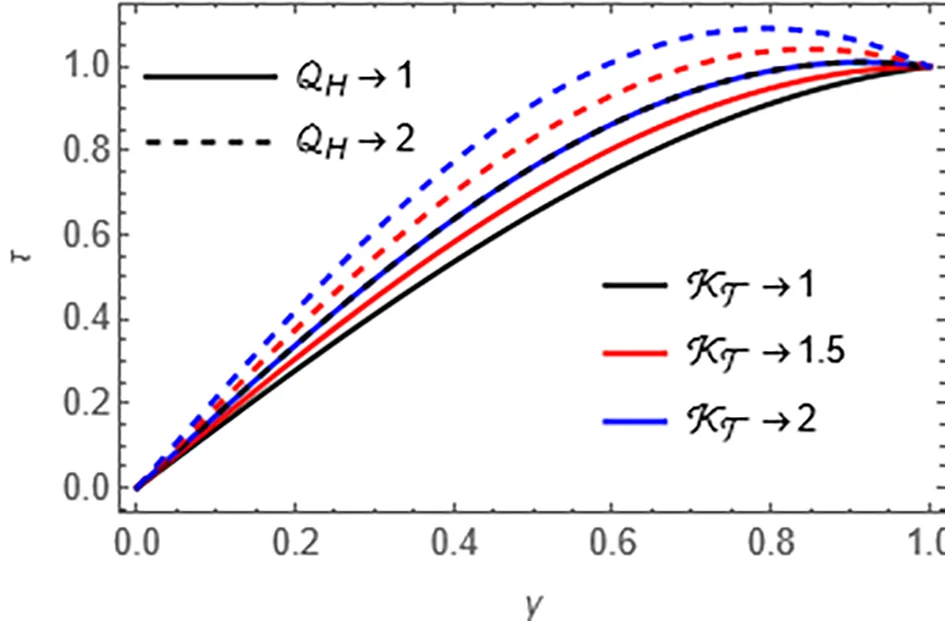
Temperature graph for values $K_{T}$ and $Q_{H}$ with ω=1,Pe=0.7 .

**Figure 3.**
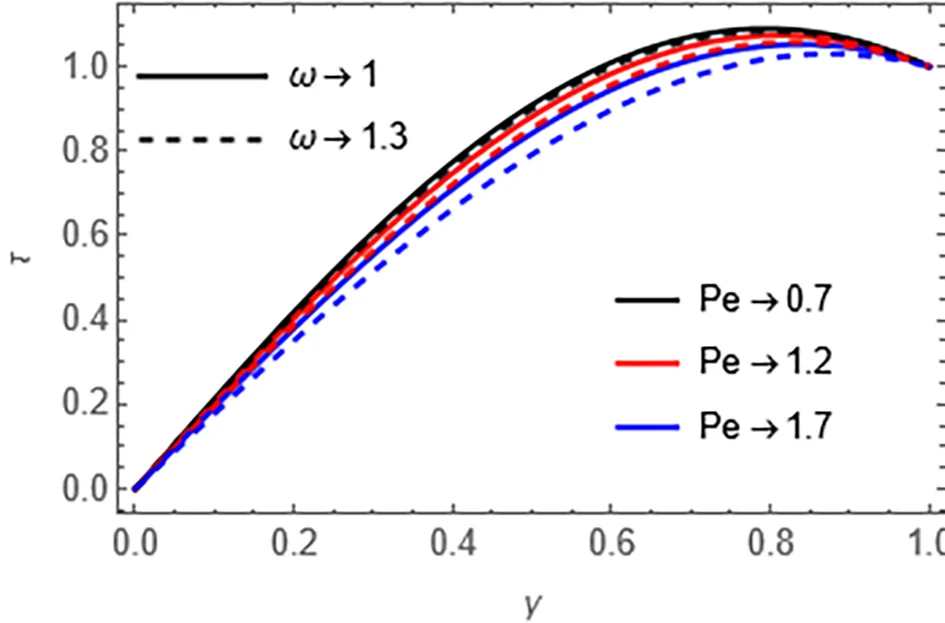
Temperature graph for values Pe and ω with $K_{T}=2,Q_{H}=2$ .

**Figure 4.**
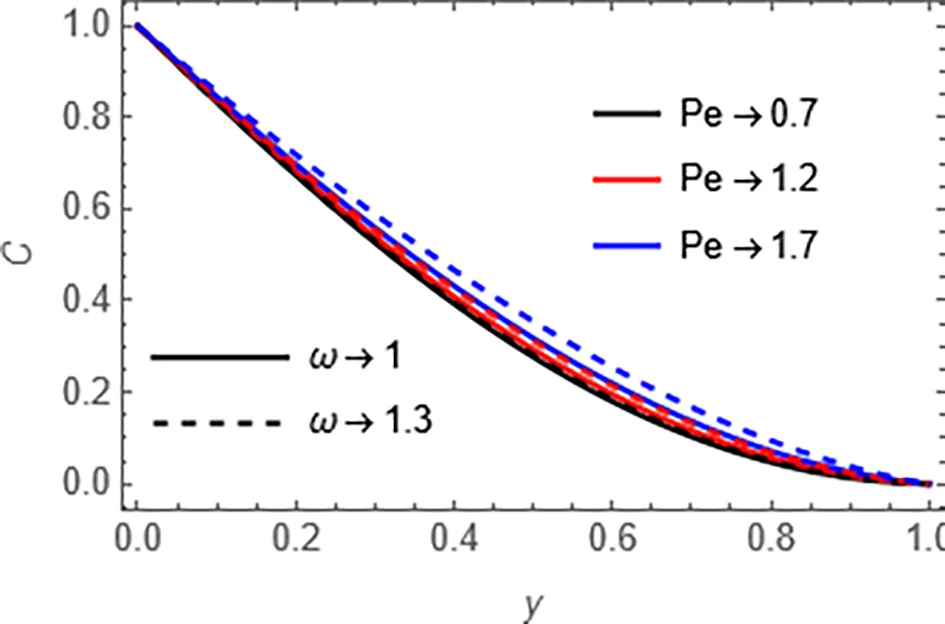
Concentration graph for values Pe and ω with $K_{T}=Q_{H}=2,S_{C}=S_{T}=K_{C}=0.7$ .

**Figure 5.**
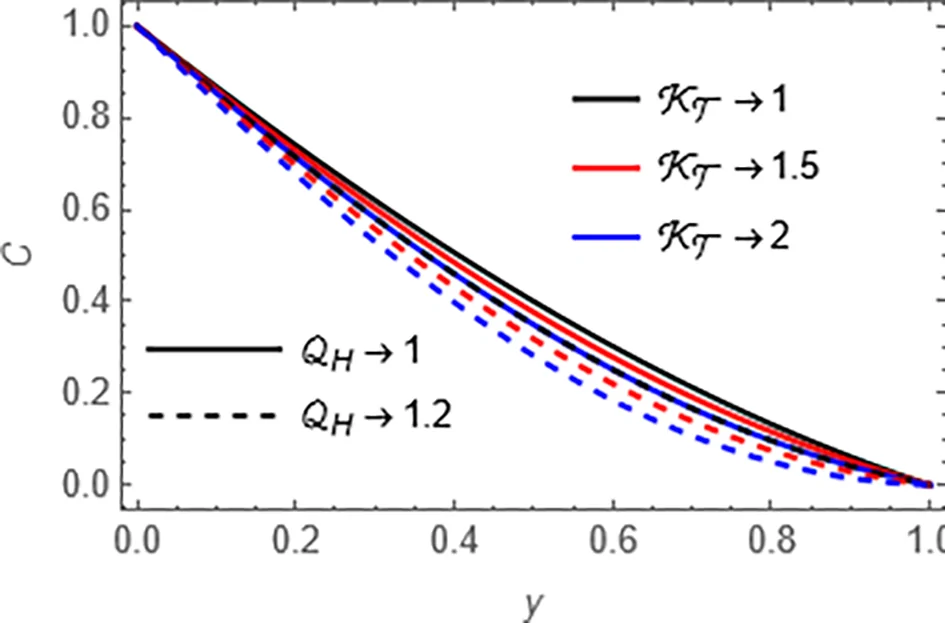
Concentration graph for values $K_{T}$ and $Q_{H}$ with $\omega =1,\mathrm{Pe}=S_{C}=S_{T}=K_{C}=0.7$ .

**Figure 6.**
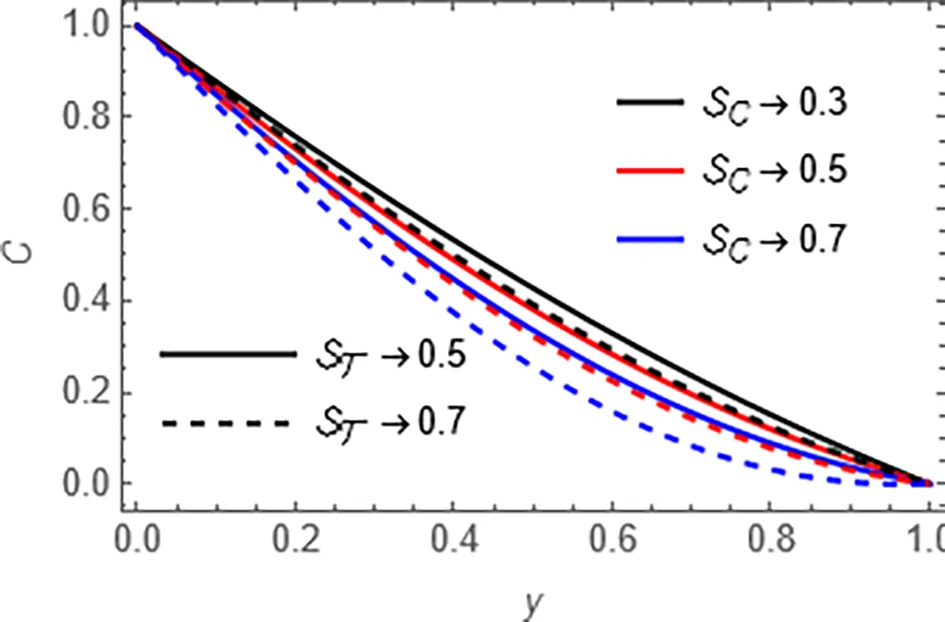
Concentration graph for values $S_{C}$ and $S_{T}$ with $K_{T}=Q_{H}=2,\omega =1,\mathrm{Pe}=K_{C}=0.7$ .

**Figure 7.**
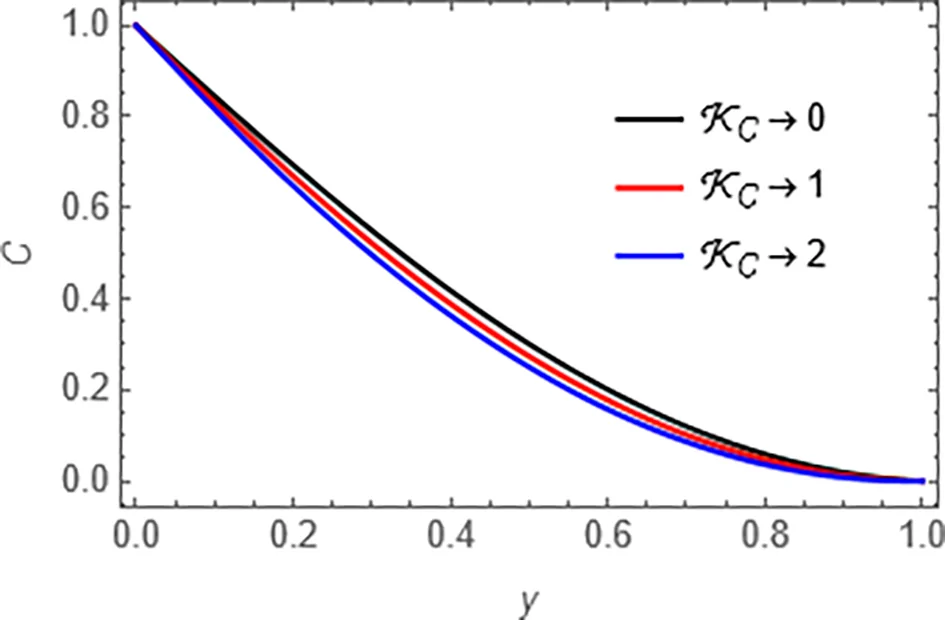
Concentration graph for values $K_{C}$ with $K_{T}=Q_{H}=2,\omega =1,\mathrm{Pe}=S_{C}=S_{T}=0.7$ .

**Figure 8.**
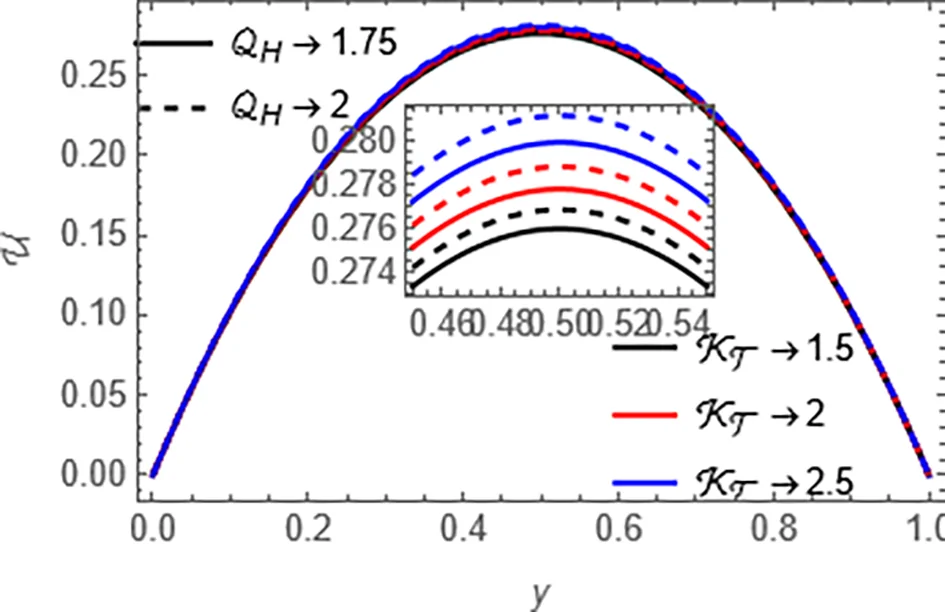
Velocity graph for values $Q_{H}$ and $K_{T}$ with $\omega =1,\lambda =0.6,S_{T}=0.3,\mathrm{Pe}=S_{C}=K_{C}=\mathrm{Da}=0.7$ , $M=1.1,G_{T}=G_{C}=0.5,F_{r}=0.8,$ $R_{e}=2,\varphi =\phi =\pi /4,\epsilon =0$ .

**Figure 9.**
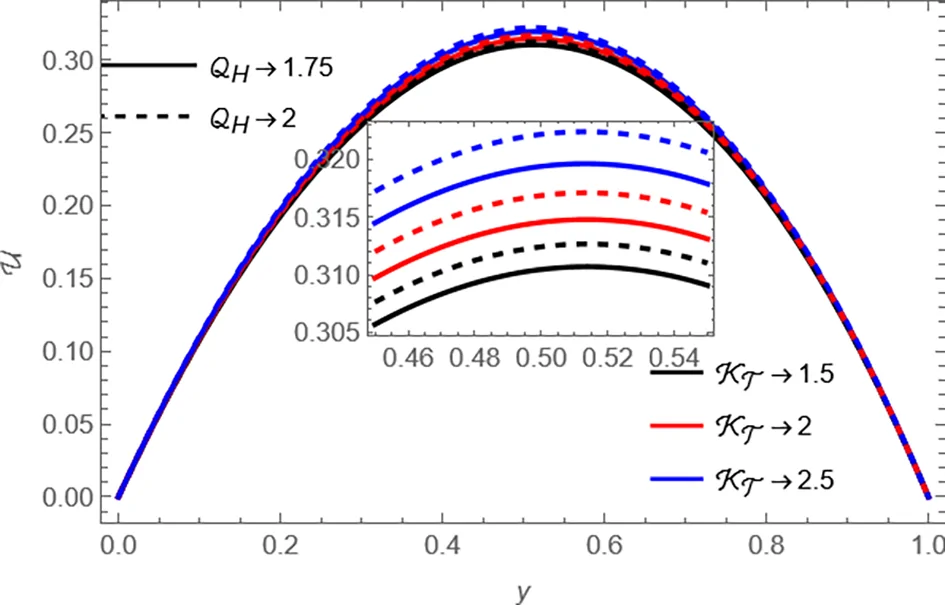
Velocity graph for values $Q_{H}$ and $K_{T}$ with $\omega =1,\lambda =0.6,S_{T}=0.3,\mathrm{Pe}=S_{C}=K_{C}=\mathrm{Da}=0.7$ , $M=1.1,G_{T}=G_{C}=0.5,F_{r}=0.8,$ $R_{e}=2,\varphi =\phi =\pi /4,\epsilon =0.2$ .

**Figure 10.**
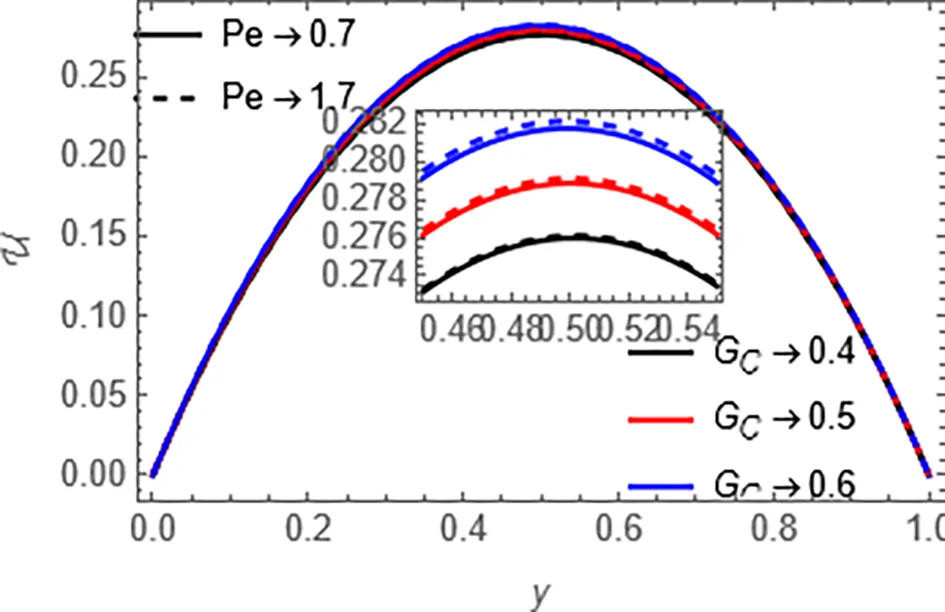
Velocity graph for values Pe and $G_{C}$ with $K_{T}=Q_{H}=2,\omega =1,\lambda =0.6,S_{T}=0.3,S_{C}=K_{C}=\mathrm{Da}=0.7$ , $M=1.1,G_{T}=0.5,F_{r}=0.8,$ $R_{e}=2,\varphi =\phi =\pi /4,\epsilon =0$ .

**Figure 11.**
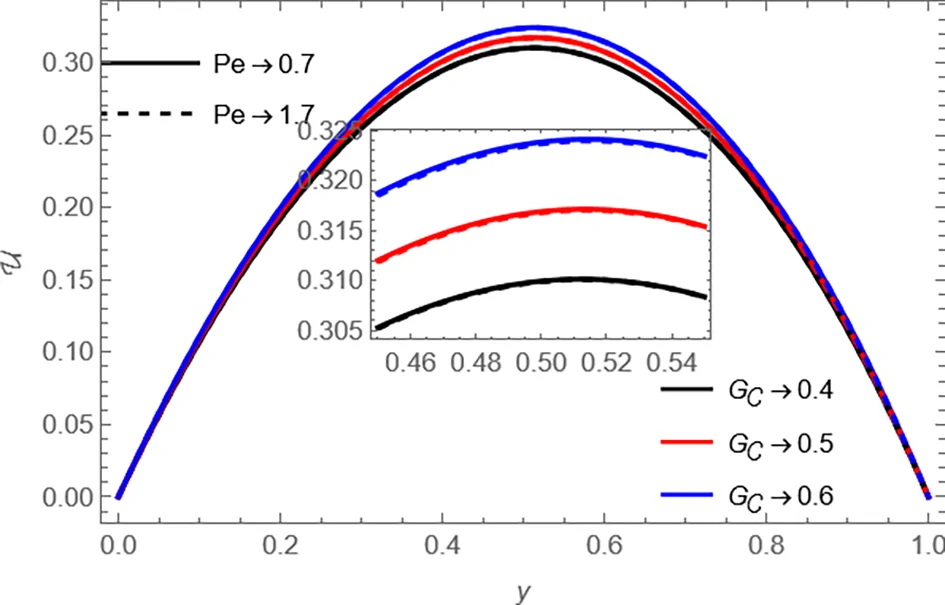
Velocity graph for values Pe and $G_{C}$ with $K_{T}=Q_{H}=2,\omega =1,\lambda =0.6,S_{T}=0.3,S_{C}=K_{C}=\mathrm{Da}=0.7$ , $M=1.1,G_{T}=0.5,F_{r}=0.8,$ $R_{e}=2,\varphi =\phi =\pi /4,\epsilon =0.2$ .

**Figure 12.**
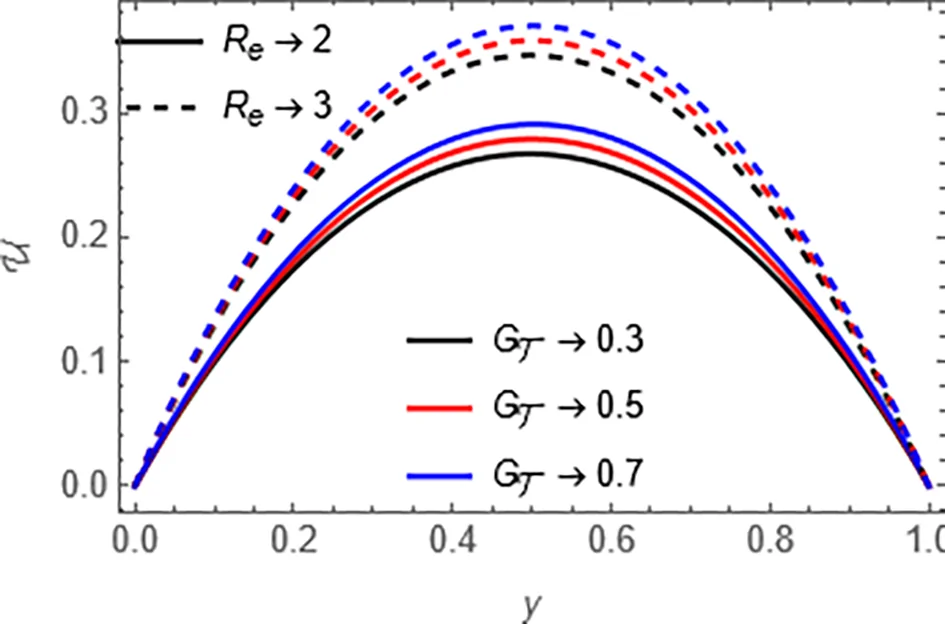
Velocity graph for values $R_{e}$ and $G_{T}$ with $K_{T}=Q_{H}=2,\omega =1,\lambda =0.6,S_{T}=0.3,\mathrm{Pe}=S_{C}=K_{C}=\mathrm{Da}=0.7$ , $M=1.1,G_{C}=0.5,F_{r}=0.8,\varphi =\phi =\pi /4,\epsilon =0$ .

**Figure 13.**
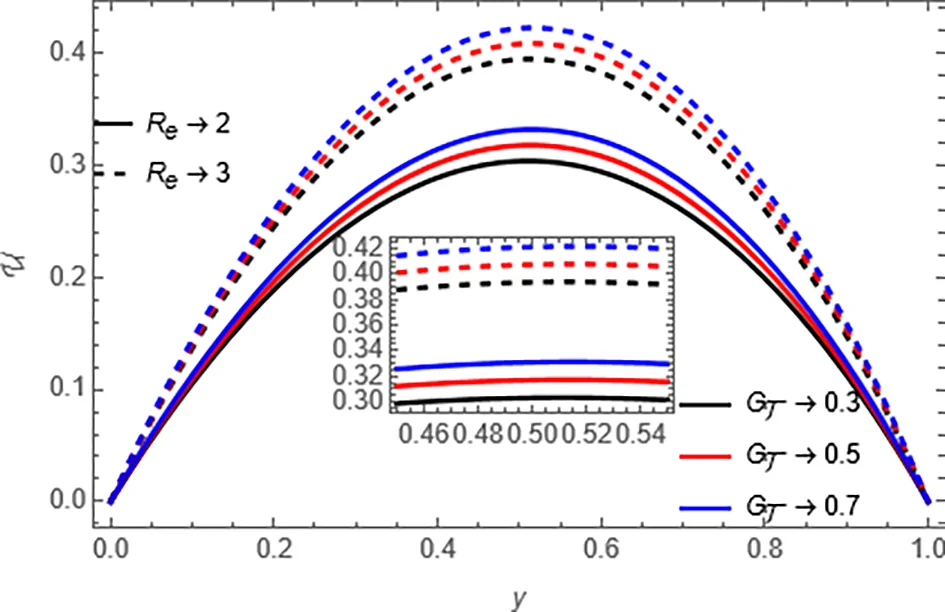
Velocity graph for values $R_{e}$ and $G_{T}$ with $K_{T}=Q_{H}=2,\omega =1,\lambda =0.6,S_{T}=0.3,\mathrm{Pe}=S_{C}=K_{C}=\mathrm{Da}=0.7$ , $M=1.1,G_{C}=0.5,F_{r}=0.8,\varphi =\phi =\pi /4,\epsilon =0.2$ .

**Figure 14.**
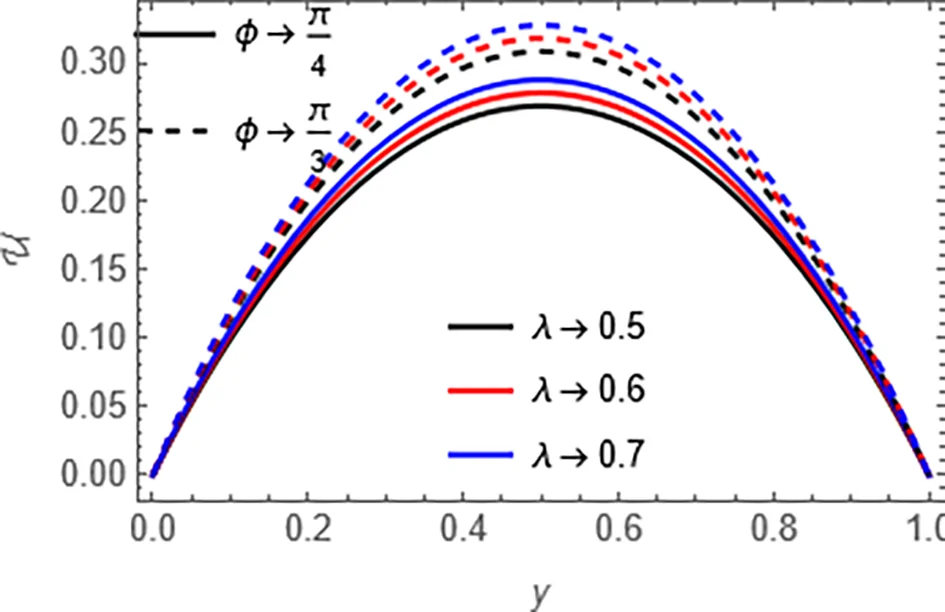
Velocity graph for values λ and ϕ with $K_{T}=Q_{H}=2,\omega =1,S_{T}=0.3,\mathrm{Pe}=S_{C}=K_{C}=\mathrm{Da}=0.7$ , $M=1.1,G_{T}=G_{C}=0.5,F_{r}=0.8,R_{e}=2,\varphi =\pi /4,\epsilon =0$ .

**Figure 15.**
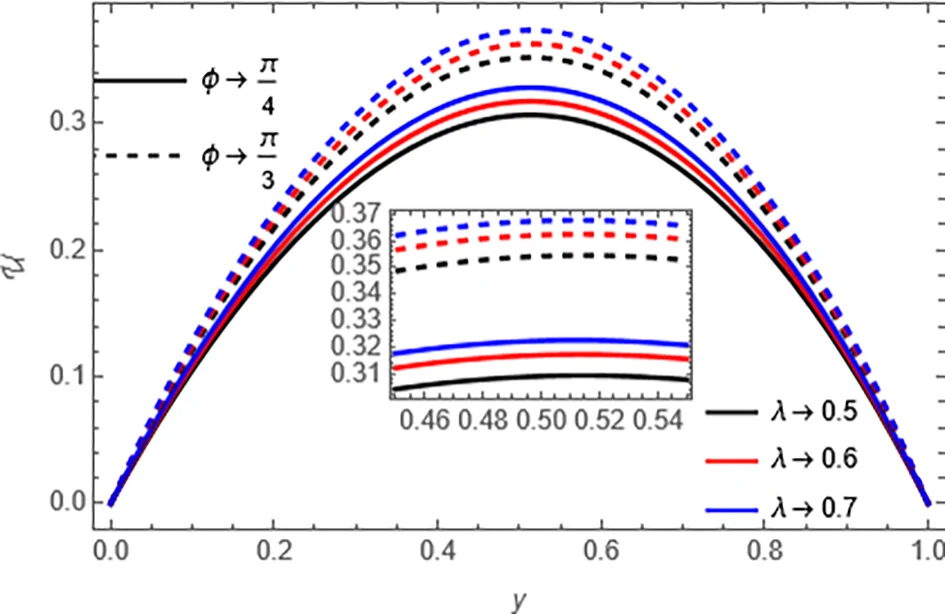
Velocity graph for values λ and ϕ with $K_{T}=Q_{H}=2,\omega =1,S_{T}=0.3,\mathrm{Pe}=S_{C}=K_{C}=\mathrm{Da}=0.7$ , $M=1.1,G_{T}=G_{C}=0.5,F_{r}=0.8,R_{e}=2,\varphi =\pi /4,\epsilon =0.2$ .

**Figure 16.**
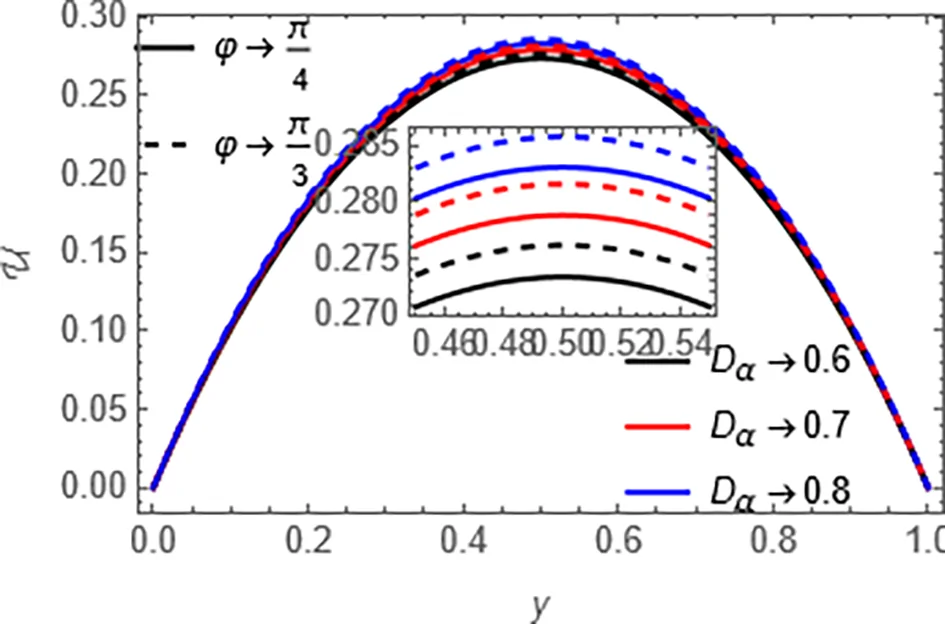
Velocity graph for values Da and φ with $K_{T}=Q_{H}=2,\omega =1,\lambda =0.6,S_{T}=0.3,\mathrm{Pe}=S_{C}=K_{C}=0.7$ , $M=1.1,G_{T}=G_{C}=0.5,F_{r}=0.8,R_{e}=2,\phi =\pi /4,\epsilon =0$ .

**Figure 17.**
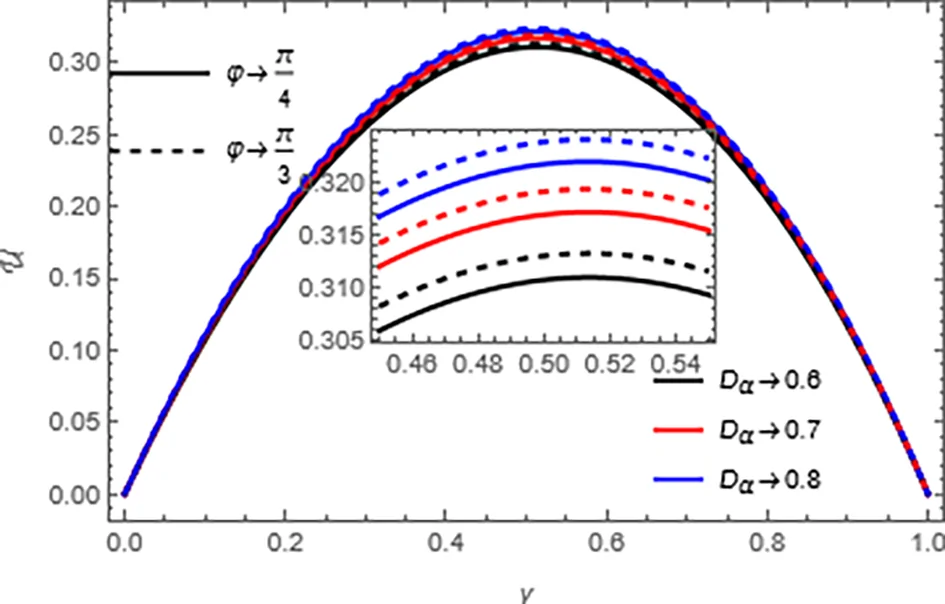
Velocity graph for values Da and φ with $K_{T}=Q_{H}=2,\omega =1,\lambda =0.6,S_{T}=0.3,\mathrm{Pe}=S_{C}=K_{C}=0.7$ , $M=1.1,G_{T}=G_{C}=0.5,F_{r}=0.8,R_{e}=2,\phi =\pi /4,\epsilon =0.2$ .

**Figure 18.**
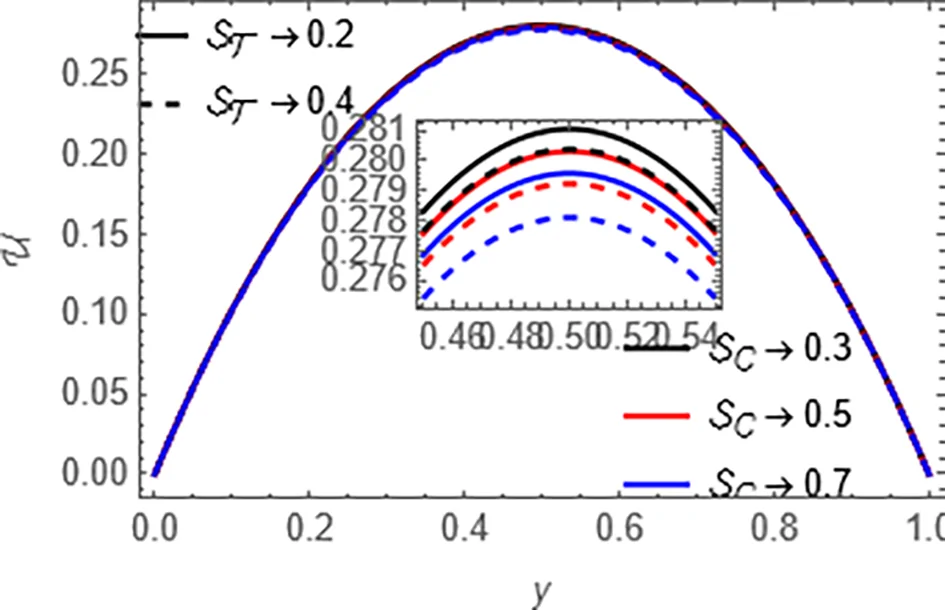
Velocity graph for values $S_{C}$ and $S_{T}$ with $K_{T}=Q_{H}=2,\omega =1,\lambda =0.6,\mathrm{Pe}=K_{C}=\mathrm{Da}=0.7$ , $M=1.1,G_{T}=G_{C}=0.5,F_{r}=0.8,R_{e}=2,\varphi =\phi =\pi /4,\epsilon =0$ .

**Figure 19.**
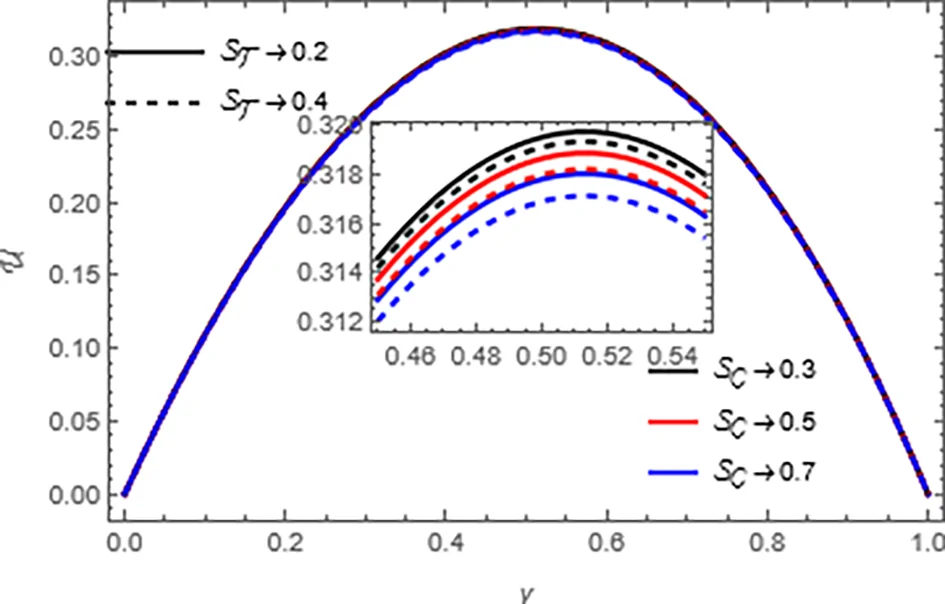
Velocity graph for values $S_{C}$ and $S_{T}$ with $K_{T}=Q_{H}=2,\omega =1,\lambda =0.6,\mathrm{Pe}=K_{C}=\mathrm{Da}=0.7$ , $M=1.1,G_{T}=G_{C}=0.5,F_{r}=0.8,R_{e}=2,\varphi =\phi =\pi /4,\epsilon =0.2$ .

**Figure 20.**
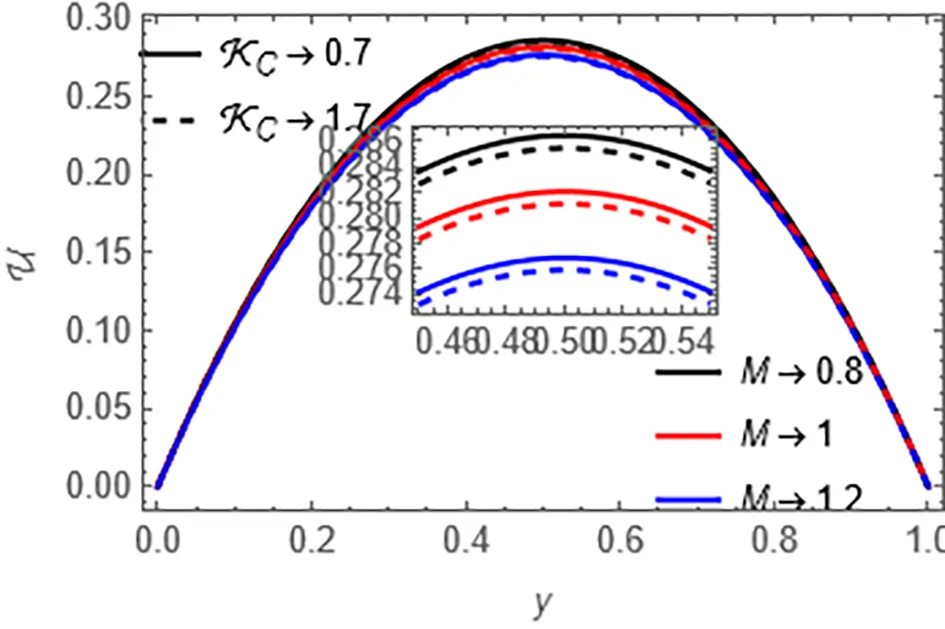
Velocity graph for values $K_{C}$ and M with $K_{T}=Q_{H}=2,\omega =1,\lambda =0.6,S_{T}=0.3,$ $\mathrm{Pe}=S_{C}=\mathrm{Da}=0.7$ , $G_{T}=G_{C}=0.5,F_{r}=0.8,$ $R_{e}=2,\varphi =\phi =\pi /4,\epsilon =0$ .

**Figure 21.**
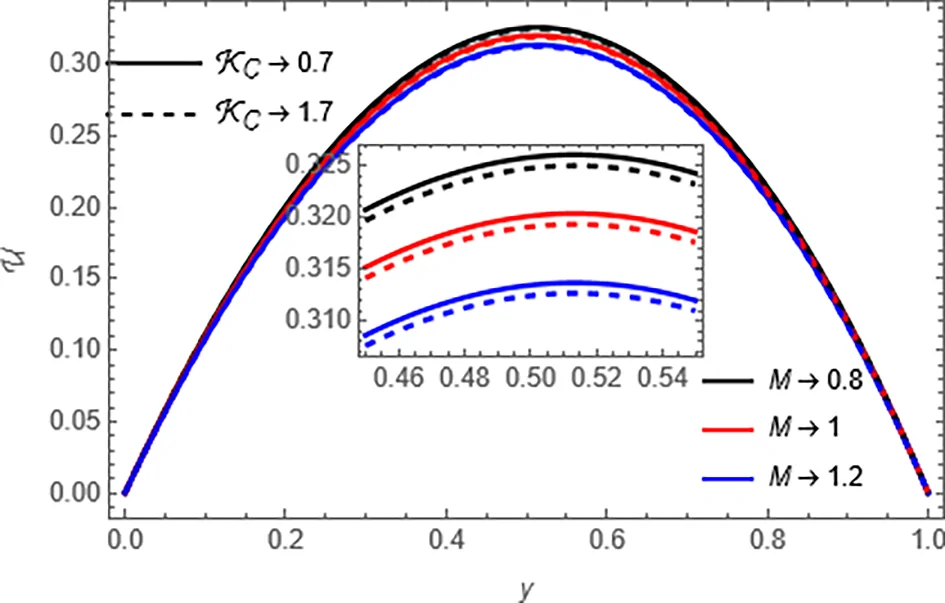
Velocity graph for values $K_{C}$ and M with $K_{T}=Q_{H}=2,\omega =1,\lambda =0.6,S_{T}=0.3,$ $\mathrm{Pe}=S_{C}=\mathrm{Da}=0.7$ , $G_{T}=G_{C}=0.5,F_{r}=0.8,$ $R_{e}=2,\varphi =\phi =\pi /4,\epsilon =0.2$ .

**Figure 22.**
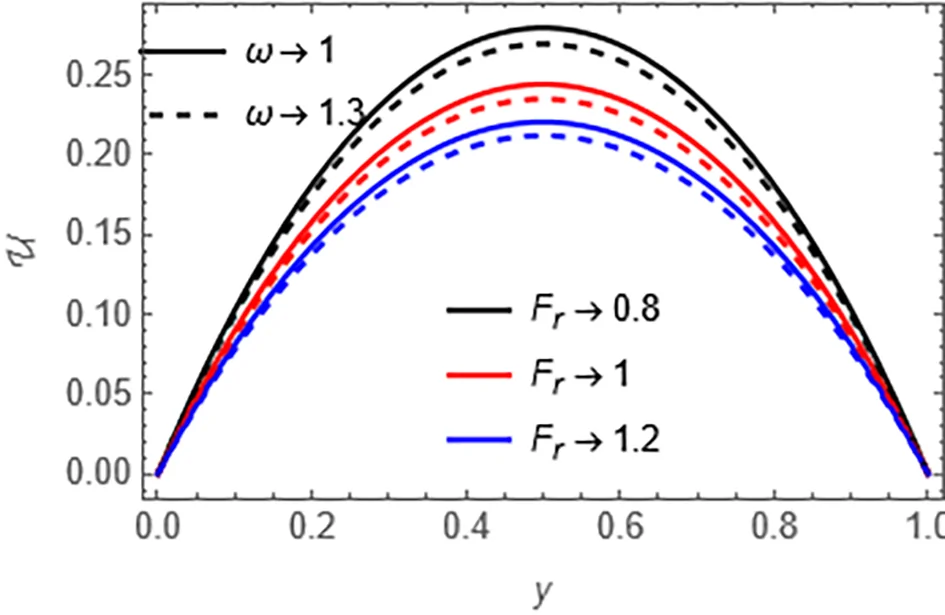
Velocity graph for values $F_{r}$ and ω with $K_{T}=Q_{H}=2,\lambda =0.6,S_{T}=0.3,\mathrm{Pe}=S_{C}=K_{C}=\mathrm{Da}=0.7$ , $\;M=1.1,G_{T}=G_{C}=0.5,$ $R_{e}=2,\varphi =\phi =\pi /4,\epsilon =0$ .

**Figure 23.**
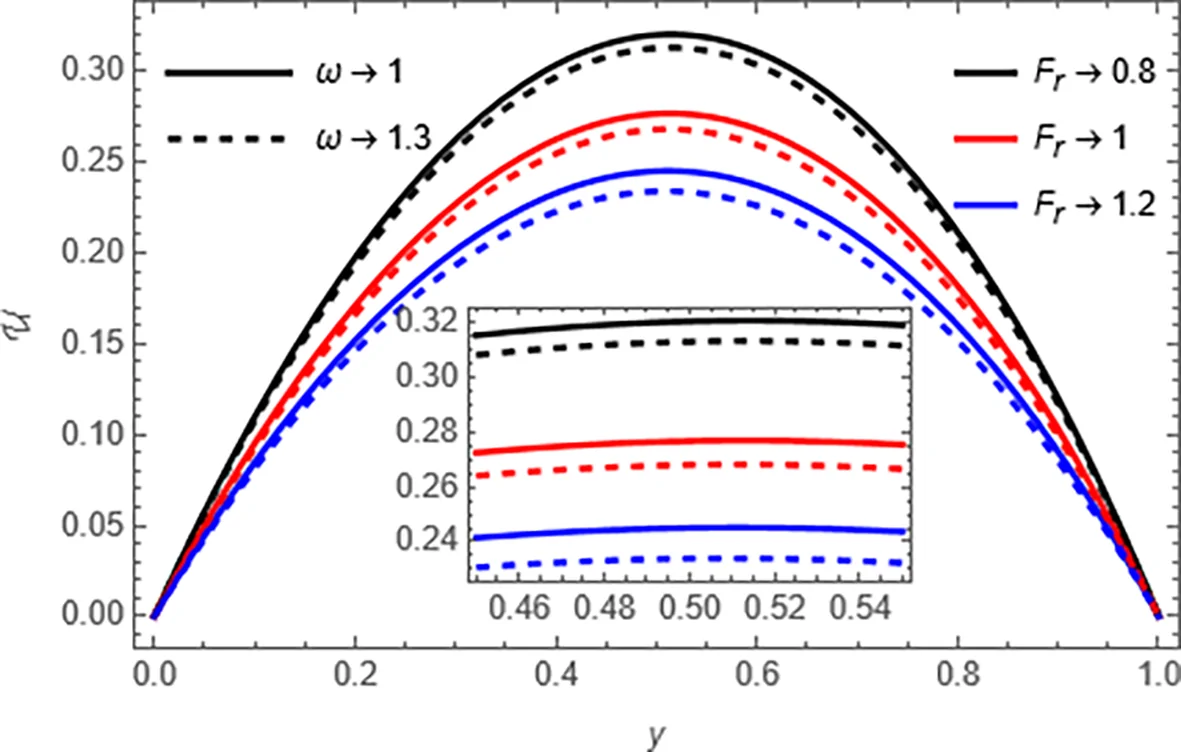
Velocity graph for values $F_{r}$ and ω with $K_{T}=Q_{H}=2,\lambda =0.6,S_{T}=0.3,\mathrm{Pe}=S_{C}=K_{C}=\mathrm{Da}=0.7$ , $\;M=1.1,G_{T}=G_{C}=0.5,$ $R_{e}=2,\varphi =\phi =\pi /4,\epsilon =0.2$ .

**Figure 24.**
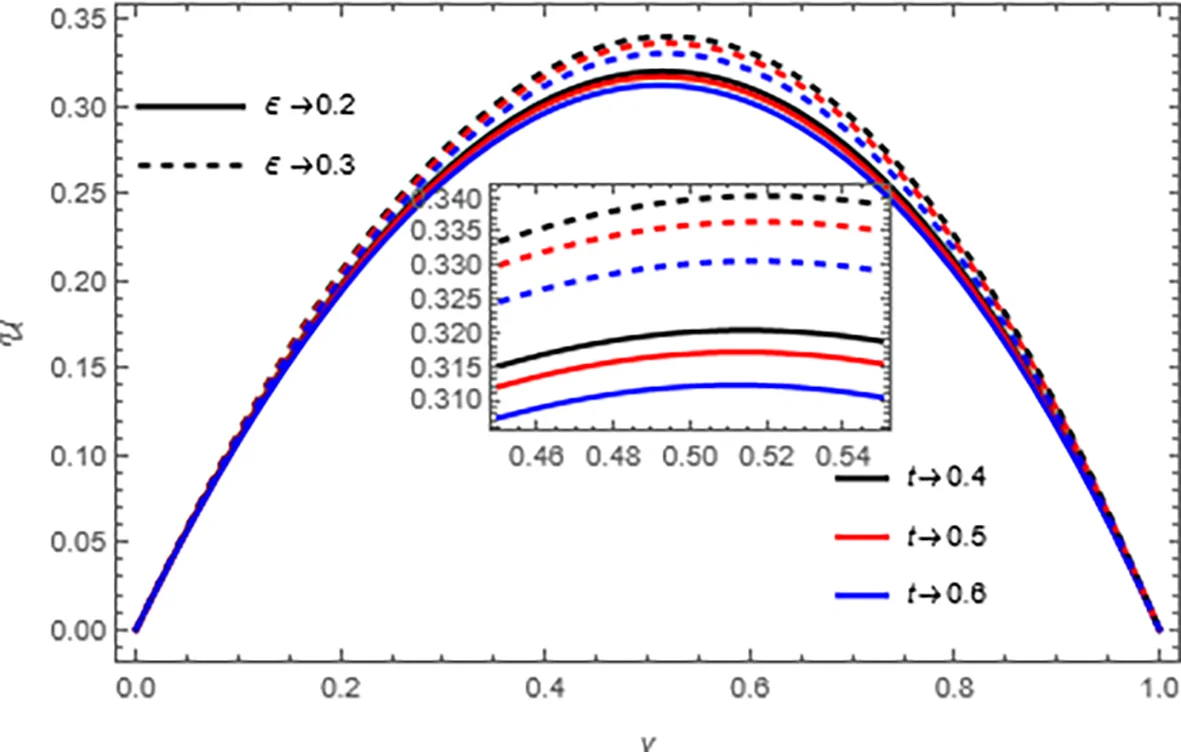
Velocity graph for values t and ϵ with $K_{T}=Q_{H}=2,\omega =1,\lambda =0.6,S_{T}=0.3,$ $\mathrm{Pe}=K_{C}=S_{C}=\mathrm{Da}=0.7$ , $G_{T}=G_{C}=0.5,F_{r}=0.8,$ $R_{e}=2,\varphi =\phi =\pi /4,M=1.1$ .

## 4. Solution analysis

This part includes analyzing the solutions we obtained. We start with the temperature function Figures 2 and 3, then the concentration function Figures 4– 7, and then the fluid velocity function Figures 8– 24.

Figure 2 The data indicates that the temperature of the fluid rises as $Q_{H}$ and $K_{T}$ increase. In contrast, Figure 3 shows that temperature fluid decreases by increasing ω and Pe .

Figures 4– 7, Fluid concentration rises with higher values of ω and *Pe*, but decreases with increased $Q_{H}$ , $K_{T}$ , $S_{C}$ , $S_{T}$ , and $K_{C}$ .

Figures 8– 24, Exhibit that fluid velocity escalates with an increase in $Q_{H}$ , $K_{T}$ , $G_{C}$ , Pe , $R_{e}$ , $G_{T}$ , λ **,** ϕ , φ , and Da , while diminishing with an increase in $S_{C}$ , $S_{T}$ , $K_{C}$ , M , ω , and $F_{r}$ . It is evident that the velocity fluctuates more significantly in the scenario of changing viscosity compared to that of constant viscosity.

## 5. Discussions and conclusion

This study analyzed the transient, incompressible magnetohydrodynamic (MHD) oscillatory flow of a non-Newtonian Bingham fluid through an inclined porous channel, considering variable viscosity, heat generation, and concentration effects. The governing nonlinear equations were solved using the separation of variables technique with computational assistance from Wolfram Mathematica 13. The obtained results were interpreted in terms of the influence of several dimensionless parameters on temperature, concentration, and velocity distributions.

• The velocity of the fluid was observed to increase with larger values of the solutal Grashof number $G_{C}$ , Reynolds number Re , thermal Grashof number $G_{T}$ , and pressure gradient constant λ . In contrast, the velocity decreased when the magnetic parameter M , and Froude number Fr were increased. Moreover, the fluid motion was enhanced with a higher channel inclination angle *ϕ*, as this promotes gravitational acceleration along the channel wall, whereas the effect of the magnetic field inclination *φ* was relatively minor.
• Increasing the Schmidt number Soret number $S_{T}\;$ and chemical reaction parameter $K_{C}$ resulted in a reduction in concentration, which in turn caused a slight decline in fluid velocity. These trends confirm the coupling between mass diffusion and flow resistance within the porous medium.
• The fluid temperature and velocity rose with larger heat generation $\;Q_{H}$ and radiation $K_{T}$ parameters, as internal heating reduces viscosity and promotes stronger convective motion. Conversely, higher values of the oscillation frequency ω and Péclet number Pe led to a decrease in both temperature and velocity, accompanied by an increase in concentration. This behavior indicates that the influence of temperature on flow acceleration is more dominant than that of concentration.
• The explicit inclusion of the Bingham number ( $B_{n}$ ) and the adoption of the Papanastasiou regularization substantially improved the physical fidelity of the model. The revised formulation accurately represents yield-stress effects, capturing both yielded and unyielded (plug) regions within the channel. Increasing $B_{n}$ expands the plug zone near the channel center, while reducing velocity gradients near the walls. These effects become more prominent under higher yield stress or reduced shear conditions, consistent with classical results (Vajravelu et al., 1987; Lakshminarayana et al., 2018). Additionally, it was observed that thermal softening mitigates yield resistance, indicating that elevated temperature partially offsets the retarding influence of yield stress. This refinement enhances the realism of the current Bingham fluid model and aligns it closely with physical flow behavior.
• An increase in the viscosity variation parameter ( ε ) amplified the velocity magnitude, particularly near the channel center, signifying that thermal dependence of viscosity plays a critical role in controlling the flow field.
• In summary, the present analysis demonstrates that the combined effects of variable viscosity, temperature, concentration, and yield stress govern the dynamic behavior of MHD oscillatory Bingham fluid flow through inclined porous channels. The inclusion of radiation and heat generation parameters provides a more comprehensive understanding of heat and mass transfer mechanisms relevant to industrial, biological, and geophysical transport processes. The extended formulation incorporating the Bingham number and yield-surface representation ensures a more robust and physically accurate model for predicting complex non-Newtonian flow phenomena.

### Physical interpretation of the governing effects

The observed flow, thermal, and concentration behaviors can be physically interpreted through the balance between the driving and resisting mechanisms acting in the inclined porous channel. The motion of the fluid is mainly driven by buoyancy forces arising from temperature and concentration differences. These buoyancy effects promote mixed convection and tend to accelerate the flow along the channel. However, this driving mechanism is opposed by several resistance effects, including the Lorentz force due to the applied magnetic field, the drag force caused by the porous medium, the viscous resistance associated with variable viscosity, and the yield-stress resistance of the Bingham fluid.

The magnetic field produces a Lorentz force that acts opposite to the motion of the electrically conducting fluid. Therefore, increasing the magnetic parameter strengthens electromagnetic damping and suppresses the velocity field. In contrast, increasing the thermal or solutal buoyancy contribution enhances the driving force and increases the fluid motion. Hence, the final velocity distribution results from the competition between buoyancy-induced acceleration and magnetic damping.

The Bingham fluid behavior introduces an additional resistance through the yield stress. Since a Bingham fluid does not deform until the applied stress exceeds a critical yield value, increasing the Bingham parameter reduces the ability of the fluid to move freely. This effect becomes more significant when the driving buoyancy force is not strong enough to overcome the yield-stress resistance. Similarly, the porous medium acts as a momentum sink because the solid matrix imposes drag on the moving fluid. As the porous resistance increases, the flow weakens and the velocity decreases.

Variable viscosity further modifies the momentum transport. When viscosity decreases with temperature, the warmer fluid layers become less resistant to shear motion, which enhances the velocity. Conversely, higher viscosity increases the internal friction between adjacent layers and reduces the flow intensity. Therefore, the flow structure is controlled by the combined influence of viscosity variation, yield stress, porous resistance, magnetic damping, and buoyancy forces.

The temperature field is governed by the interaction between thermal diffusion and convective heat transport. When the velocity is enhanced, convective heat transport becomes stronger, allowing heat to be redistributed more effectively through the channel. However, when the magnetic field, porous resistance, or yield stress suppresses the velocity, heat transfer becomes more conduction-dominated. In the same way, the concentration field depends on the balance between molecular diffusion and convective mass transport. Stronger fluid motion enhances species transport, whereas flow suppression causes weaker mass transfer and stronger concentration gradients.

Overall, the results show that the behavior of the system cannot be attributed to a single parameter alone. Instead, the velocity, temperature, and concentration profiles are controlled by the coupled competition between buoyancy forces, Lorentz-force damping, Bingham yield stress, variable viscosity, porous-medium resistance, and thermal and solutal diffusion mechanisms.

### Computational procedure and reproducibility

• Software: *Wolfram Mathematica 13*;
• Method: symbolic + numerical integration of the ODE system;
• Mesh size = 500 points, tolerance = 10 ^−8^, maximum residual < 10 ^−5^;
• Convergence criteria and supporting computational materials are provided as extended data in the public repository (see Data Availability section).
• All parameter values used for figures provided.

## Data Availability

No underlying data are associated with this article. This study is theoretical and computational, and numerical values used to generate all figures are produced dynamically using the Mathematica code provided as extended data. Zenodo: Data and code for: Effects of variable viscosity, concentration and heat variation on MHD oscillatory flow for Bingham fluid through an inclined porous channel. https://doi.org/10.5281/zenodo.18300187 ^37^ This project contains the following extended data:
• 1-Velocity.nb – Mathematica code for velocity profiles (case I). • 2-Velocity.nb – Mathematica code for velocity profiles (case II). • Temp.nb – Mathematica code for temperature distributions. • Conce.nb – Mathematica code for concentration distributions. Data are available under the terms of the Creative Commons Attribution 4.0 International (CC-BY 4.0) license.
