# Supplementary material for: Effects of variable viscosity, concentration and heat variation on MHD oscillatory flow for Bingham fluid through an inclined porous channel
Source: F1000Res. 2026 Jun 23;15:476. Originally published 2026 Apr 6. [Version 2] doi: 10.12688/f1000research.172909.2 (PMC13602403; doi:10.12688/f1000research.172909.2)
Supplement: Peer review history

# Reviewer report: Reviewer response for version 2

**DOI:** 10.5256/f1000research.201751.r496492

**Published:** 2026-07-11

**Authors:** Lakshminarayana P

**Affiliations:** 1 Vellore Institute of Technology, Vellore, Tamil Nadu, India

This study investigates the effects of variable viscosity, concentration, and heat transfer on the magnetohydrodynamic (MHD) oscillatory flow of a Bingham fluid through an inclined porous channel. The influence of key physical parameters on the velocity, temperature, and concentration distributions is analyzed to better understand the transport characteristics of non-Newtonian fluids in porous media. Below, I have outlined some constructive feedback and recommendations to improve the manuscript.

1. The abstract does not mention the practical applications of the present study. The authors are requested to include the engineering and/or real-world applications of the proposed model in the abstract.
2. The introduction relies on several older references. It is recommended to incorporate more recent and relevant literature to better reflect the current state of research.
3. Appropriate references should be provided for the governing equations to establish the validity and reliability of the mathematical formulation.
4. The manuscript should clearly specify whether the porous medium is distributed throughout the channel or is confined to the channel walls.
5. How this study is different from the following recent studies. Authors may include the discussion in the introduction to strengthen the background on magnetohydrodynamics, inclined channels, and buoyancy effects: Magnetohydrodynamic Peristaltic Flow of Hybrid Nanofluid in an Asymmetric Channel with Thermal Radiation and Shape Factors: An Application to Coolant Systems; Convective peristaltic pumping of MHD Ree-Eyring nanofluid in a chemically reacting flexible divergent channel with activation energy and radiation; Investigation of a conducting Casson fluid flow through a porous flexible microfluidic channel with catalytic effects: application in pharmaceutical fluid processing; Cilia-Mediated Electroosmotic Magnetohydrodynamic Hybrid Nanofluid Transport for Biomedical Applications: Impact of Radiation and Nanoparticle Geometry.
6. To better highlight the novelty of the present work, a comparison (novelty) table should be included at the end of the introduction, clearly distinguishing the proposed study from previously published works.
7. The figures should include directional arrows indicating the influence (increasing or decreasing trends) of the governing parameters on the flow characteristics to improve clarity and readability.
8. The basis for selecting the ranges of the governing parameters should be clearly justified, preferably with appropriate references or practical considerations.
9. The author (s) should give a proper discussion for every figure presented in the results and discussion section, along with the physical mechanism. Use the arrow in the figures for a better understanding of the trend of the given parameter on the flow. Please see the refs: Diffusion effects on mixed convective peristaltic flow of a bi-viscous Bingham nanofluid through a porous medium with convective boundary conditions; Energy Diffusion Efficiency through Peristaltic–Electroosmotic Flow of (Cu–TiO₂–GO)/H₂O Trihybrid Nanofluid in Sterilization and Pasteurization: Application to Food Processing; Bioadaptive peristaltic model of double-diffusive nanofluid through a slant porous channel: application to hazardous compound mitigation;
10. A detailed physical interpretation should be provided for the effects of all governing parameters discussed in the results section.
11. The manuscript should present separate sections for Results and Discussion and Conclusion to improve its organization and readability.
12. A dedicated discussion on the limitations of the present study and possible directions for future research should be included.
13. The numerical or analytical results should be validated through comparison with previously published results or established benchmark solutions.
14. A comprehensive nomenclature section should be included, listing all symbols, abbreviations, and their corresponding SI units.

Is the work clearly and accurately presented and does it cite the current literature?

Partly

If applicable, is the statistical analysis and its interpretation appropriate?

Not applicable

Are all the source data underlying the results available to ensure full reproducibility?

Partly

Is the study design appropriate and is the work technically sound?

Yes

Are the conclusions drawn adequately supported by the results?

Yes

Are sufficient details of methods and analysis provided to allow replication by others?

Yes

Reviewer Expertise:

Fluid dynamics; Heat transfer, MHD; Boundary layer theory; Peristalsis; Flow through Porous media.

I confirm that I have read this submission and believe that I have an appropriate level of expertise to confirm that it is of an acceptable scientific standard, however I have significant reservations, as outlined above.

# Reviewer report: Reviewer response for version 2

**DOI:** 10.5256/f1000research.201751.r496483

**Published:** 2026-07-10

**Authors:** Ali J. Chamkha

**Affiliations:** 1 Kuwait College of Science and Technology, Doha, Kuwait

The paper is a revised version that this reviewer did not review before. The paper is good but can be improved. Therefore, the paper can be recommended for indexing provided that the authors address the following comments:

1. Add a Nomenclature.
2. The paper can benefit from editing the English language throughout.
3. The literature review is good but can be improved. The following related work can be checked and considered: Hydromagnetic natural convection from an inclined porous square enclosure with heat generation.
4. Discuss a real application for this problem with the many included effects.
5. Justify why the convective terms in the equations are neglected.
6. Based on the order of magnitudes analysis, explain why the magnetic, porous resistance and the viscous dissipations are not included in this study.
7. Justify the neglection of the induced magnetic field and Hall effect. See for instance, the following related work: Hall effects on mhd peristaltic flow of Jeffrey fluid through porous medium in a vertical stratum; Unsteady flow and heat transfer on a semi-infinite flat plate with an aligned magnetic field.
8. State the condition for which the Forchheimer inertia effect of the porous media becomes important and justify its neglection. See for example, Darcy-Forchheimer relation in Casson type MHD nanofluid flow over non-linear stretching surface.
9. Justify the assumption of local thermal equilibrium between the fluid and the porous media in one region.
10. Why is the porosity of the media not included in the equations? Please explain.
11. Comparisons with previously published work should be made to ensure correctness of the closed-form solutions.
12. On what basis are the parametric values chosen? Do they correspond to a specific physical condition?

Is the work clearly and accurately presented and does it cite the current literature?

Yes

If applicable, is the statistical analysis and its interpretation appropriate?

Not applicable

Are all the source data underlying the results available to ensure full reproducibility?

Yes

Is the study design appropriate and is the work technically sound?

Yes

Are the conclusions drawn adequately supported by the results?

Yes

Are sufficient details of methods and analysis provided to allow replication by others?

Yes

Reviewer Expertise:

Thermo-fluid Sciences

I confirm that I have read this submission and believe that I have an appropriate level of expertise to confirm that it is of an acceptable scientific standard.

# Reviewer report: Reviewer response for version 2

**DOI:** 10.5256/f1000research.201751.r496026

**Published:** 2026-07-01

**Authors:** Oluwole Daniel Makinde

**Affiliations:** 1 Stellenbosch University, Cape Town, South Africa

The revised version seems okay.

Is the work clearly and accurately presented and does it cite the current literature?

Partly

If applicable, is the statistical analysis and its interpretation appropriate?

Not applicable

Are all the source data underlying the results available to ensure full reproducibility?

Yes

Is the study design appropriate and is the work technically sound?

Partly

Are the conclusions drawn adequately supported by the results?

Yes

Are sufficient details of methods and analysis provided to allow replication by others?

Yes

Reviewer Expertise:

Fluid Mechanics, Thermal Sciences, MHD, Mathematical modelling, Heat and Mass Transfer

I confirm that I have read this submission and believe that I have an appropriate level of expertise to confirm that it is of an acceptable scientific standard.

# Reviewer report: Reviewer response for version 1

**DOI:** 10.5256/f1000research.190674.r473533

**Published:** 2026-05-07

**Authors:** Gabriella Bognár

**Affiliations:** 1 University of Miskolc, Miskolc-Egyetemváros, Hungary

The reviewer already identified a major inconsistency that ****the momentum equation and dimensionless equation do not match.  This is a fundamental problem. Correct governing equations.

 Please highlight the novel aspects of the article in the abstract and at the end of the introduction.

 The studied effects the MHD flow, Bingham fluid, variable viscosity, concentration / heat transfer

 and porous medium are all well studied in literature, many cited papers cover similar formulations.

 The physical interpretation of the results are mostly “velocity increases with parameter X”, “temperature decreases with parameter Y”. It lacks the force balance interpretation and the competition of effects of Lorentz force vs buoyancy and yield stress vs viscosity.

 The paper needs language polishing!

 Some parameters not physically explained.

 The literature is mainly descriptive. Update the introduction section.  Remove some old-fashioned studies. An updated and complete literature review should be conducted. It should appear as part of the Introduction while bearing in mind the work's taking into account the scope and the readership of the journal.

 You can consider the following: 

 Reference 1

 Reference 2

 Reference 3

 Add a comparison with literature.

Is the work clearly and accurately presented and does it cite the current literature?

Partly

If applicable, is the statistical analysis and its interpretation appropriate?

Not applicable

Are all the source data underlying the results available to ensure full reproducibility?

Yes

Is the study design appropriate and is the work technically sound?

Partly

Are the conclusions drawn adequately supported by the results?

Partly

Are sufficient details of methods and analysis provided to allow replication by others?

No

Reviewer Expertise:

Heat and mass transfer of nanofluid flows, tribology

I confirm that I have read this submission and believe that I have an appropriate level of expertise to confirm that it is of an acceptable scientific standard, however I have significant reservations, as outlined above.

## References

[1] : Numerical analysis of Marangoni convection in a tangent hyperbolic fluid with Cattaneochristov heat and mass flux under buoyancy and activation energy effects. *Results in Engineering* .2026;29: 10.1016/j.rineng.2025.108548 10.1016/j.rineng.2025.108548

[2] : Magnetohydrodynamics effect of Marangoni nano boundary layer flow and heat transfer with CNT and radiation. *Journal of Magnetism and Magnetic Materials* .2023;575: 10.1016/j.jmmm.2023.170721 10.1016/j.jmmm.2023.170721

[3] : Rheological analysis of thermodynamics and viscosity ratio impact on non-Newtonian tetra-Bingham nanofluid stagnation point flow driven by stretching plate and a circular cylinder. *International Journal of Numerical Methods for Heat & Fluid Flow* .2026;36(1) : 10.1108/HFF-08-2025-0575254-274 10.1108/HFF-08-2025-0575

# Response

**Published:** 2026-05-15

**Authors:** Munirah Alotaibi

**Affiliations:** Mathematical Sciences, Princess Nourah bint Abdulrahman University, Riyadh, Riyadh Province, Saudi Arabia

**Response to Reviewer 2**

 Thank you very much for your careful evaluation of our manuscript and for your constructive comments. We have revised the manuscript thoroughly according to the points raised.

 **Comment 1:** The reviewer already identified a major inconsistency: the momentum equation and dimensionless equation do not match. This is a fundamental problem. Correct governing equations.

 **Response:** We sincerely thank the reviewer for pointing out this important issue. We agree that consistency between the dimensional and dimensionless governing equations is essential. Therefore, the governing momentum equation has been carefully rechecked and corrected. The nondimensionalization procedure has also been revised to ensure that the associated dimensionless equation follows correctly from the dimensional model. The related equations, notation, and parameter definitions have been checked throughout the manuscript to avoid any further inconsistency.

 **Comment 2:** Please highlight the novel aspects of the article in the abstract and at the end of the Introduction.

 **Response:** Thank you for this valuable suggestion. The abstract has been revised to highlight the novelty of the study more clearly. In addition, a novelty statement has been added at the end of the Introduction. The revised text now emphasizes that the contribution of the present work lies in developing an analytical formulation for oscillatory MHD mixed convection flow of a Bingham fluid through an inclined porous channel while considering the combined effects of variable viscosity, porous-medium resistance, heat generation, radiation, concentration transport, magnetic damping, and yield-stress behavior.

 **Comment 3:** The studied effects, including MHD flow, Bingham fluid, variable viscosity, concentration/heat transfer, and porous medium, are all well studied in the literature, and many cited papers cover similar formulations.

 **Response:** We agree with the reviewer that previous studies have widely investigated these individual effects. Accordingly, the revised manuscript now clarifies that the novelty is not based on each effect individually but on their combined treatment within a single analytical oscillatory channel-flow model. The Introduction has been improved to better identify the research gap and to explain how the present formulation differs from earlier studies that considered these mechanisms separately or in different flow configurations.

 **Comment 4:** The physical interpretation of the results is mostly limited to statements such as “velocity increases with parameter X” and “temperature decreases with parameter Y.” It lacks force-balance interpretation and the competition of effects of Lorentz force versus buoyancy and yield stress versus viscosity.

 **Response:** Thank you for this important observation. The physical interpretation of the results has been strengthened in the revised manuscript. A dedicated explanation has been added to clarify the competition between the driving buoyancy forces and the resisting Lorentz force. The discussion also explains the influence of Bingham yield stress, viscosity variation, and porous-medium drag on the flow behavior. This revision provides a more mechanistic interpretation rather than only describing whether the velocity, temperature, or concentration increases or decreases with a given parameter.

 **Comment 5:** The paper needs language polishing.

 **Response:** The manuscript has been carefully revised and language-polished to improve grammar, readability, sentence structure, and scientific clarity.

 **Comment 6:** The literature is mainly descriptive. Update the Introduction section. Remove some old-fashioned studies. An updated and complete literature review should be conducted. It should appear as part of the introduction while bearing in mind the scope and readership of the journal.

 **Response:** Thank you for this helpful suggestion. The introduction has been revised to improve the literature review and make it more focused on the scope and readership of the journal. Additional recent and relevant studies have been incorporated, including the references suggested by the reviewer. We have not removed the older references because they provide useful background for the development of the topic; however, the revised introduction now better connects both earlier and recent studies to the present problem and clearly identifies the remaining research gap. We have also reduced the reliance on purely descriptive statements by linking previous studies more directly to the motivation and novelty of the present work.

 **Comment 7:** You can consider the following references: Reference 1, Reference 2, and Reference 3.

 **Response:** Thank you for suggesting these relevant studies. The recommended references have been added to the revised introduction. They have been used to strengthen the discussion of recent developments in non-Newtonian fluid flow, MHD transport, thermal radiation, rheological effects, and heat and mass transfer mechanisms.

 We sincerely thank the reviewer for these valuable comments, which have helped us improve the mathematical consistency, novelty presentation, literature background, physical interpretation, parameter explanation, and overall clarity of the manuscript.

# Reviewer report: Reviewer response for version 1

**DOI:** 10.5256/f1000research.190674.r473534

**Published:** 2026-04-28

**Authors:** Oluwole Daniel Makinde

**Affiliations:** 1 Stellenbosch University, Cape Town, South Africa

The authors presented an analytical solution to a model problem that described MHD mixed convection oscillatory flow of Bingham fluid through an inclined porous channel. The following improvement must be incorporated:

 1.) Include appropriate expressions for the skin friction, Nusselt number, and Sherwood number. Figures showing the effects of emerging parameters on skin friction, Nusselt number, and Sherwood number must be incorporated.

 2.) Include figures depicting the impact of the oscillatory nature on the velocity, temperature, and concentration profiles.

 3.) The model momentum equation (13) failed to reflect the associated dimensionless equation (17). Correct this major technical error and rework the entire manuscript.

 4.) Update the writeup with the following relevant published papers:

 Reference 1 *. Journal of Magnetism and Magnetic Materials, Vol. 594, 171848(17pages), 2024.*

 Reference 2. Journal of Applied Mechanics, Vol. 80(6), 061003, 2013.

 Reference 3 *International Journal of Ambient Energy, Vol.45(1), 2423226 (8pages), 2024.*

Is the work clearly and accurately presented and does it cite the current literature?

Partly

If applicable, is the statistical analysis and its interpretation appropriate?

Not applicable

Are all the source data underlying the results available to ensure full reproducibility?

Yes

Is the study design appropriate and is the work technically sound?

Partly

Are the conclusions drawn adequately supported by the results?

Yes

Are sufficient details of methods and analysis provided to allow replication by others?

Yes

Reviewer Expertise:

Fluid Mechanics, Thermal Sciences, MHD, Mathematical modelling, Heat and Mass Transfer

I confirm that I have read this submission and believe that I have an appropriate level of expertise to confirm that it is of an acceptable scientific standard, however I have significant reservations, as outlined above.

## References

[1] : Computational analysis of entropy generation minimization and heat transfer enhancement in magnetohydrodynamic oscillatory flow of ferrofluids. *Journal of Magnetism and Magnetic Materials* .2024;594: 10.1016/j.jmmm.2024.171848 10.1016/j.jmmm.2024.171848

[2] : Unsteady Hydromagnetic Natural Convection Flow of a Dusty Fluid Past an Impulsively Moving Vertical Plate With Ramped Temperature in the Presence of Thermal Radiation. *Journal of Applied Mechanics* .2013;80(6) : 10.1115/1.4023959 10.1115/1.4023959

[3] : Temperature-dependent density effects on oscillatory mixed convective flow across a non-conducting horizontal circular cylinder embedded in a porous medium. *International Journal of Ambient Energy* .2024;45(1) : 10.1080/01430750.2024.2423226 10.1080/01430750.2024.2423226

# Response

**Published:** 2026-05-15

**Authors:** Munirah Alotaibi

**Affiliations:** Mathematical Sciences, Princess Nourah bint Abdulrahman University, Riyadh, Riyadh Province, Saudi Arabia

Thank you very much for your careful reading of the manuscript and for your valuable comments and suggestions. We have revised the manuscript carefully in response to the points raised.

 Comment 1: Include appropriate expressions for the skin friction, Nusselt number, and Sherwood number. Figures showing the effects of emerging parameters on skin friction, Nusselt number, and Sherwood number must be incorporated.

 Response: Thank you for this important suggestion. We fully acknowledge that the skin friction coefficient, Nusselt number, and Sherwood number are useful engineering quantities for evaluating momentum, heat-transfer, and mass-transfer rates. However, the main objective of the present work is to obtain and discuss the analytical solutions for the velocity, temperature, and concentration distributions of MHD mixed convection oscillatory flow of a Bingham fluid through an inclined porous channel. Therefore, in the revised manuscript, we have kept the focus on the analytical field solutions and their physical interpretation. Adding separate expressions and additional graphical analyses for skin friction, Nusselt number, and Sherwood number would substantially extend the scope of the present study. Accordingly, these quantities are not included in the current revision and may be considered in a future extension of this work.

 Comment 2: Include figures depicting the impact of the oscillatory nature on the velocity, temperature, and concentration profiles.

 Response: Thank you for this constructive comment. The oscillatory nature of the flow has already been examined in the manuscript through the oscillation frequency parameter (\omega). Figures showing the effects of different values of (\omega) on the velocity, temperature, and concentration profiles are included (Figs. 3, 4, 22, 23).

 Comment 3: The model momentum equation (13) failed to reflect the associated dimensionless equation (17). Correct this major technical error and rework the entire manuscript.

 Response: We sincerely thank the reviewer for identifying this important issue. We agree that the inconsistency between the dimensional momentum equation and the corresponding dimensionless equation was a typographical error. The model momentum equation has now been corrected, and the nondimensionalization procedure has been carefully rechecked. The related equations, parameter definitions, notation, and discussion have also been revised to ensure consistency between the dimensional and dimensionless formulations throughout the manuscript.

 Comment 4: Update the write-up with the following relevant published papers:

 Reference 1. Journal of Magnetism and Magnetic Materials, Vol. 594, 171848, 2024.

 Reference 2. Journal of Applied Mechanics, Vol. 80(6), 061003, 2013.

 Reference 3. International Journal of Ambient Energy, Vol. 45(1), 2423226, 2024.

 Response: Thank you for suggesting these relevant references. The introduction has been updated by incorporating the recommended studies. These papers have been used to strengthen the literature background related to MHD oscillatory flow, unsteady hydromagnetic convection, porous media, heat transfer, and mixed convection. The revised introduction now better reflects recent developments and places the present work more clearly within the existing literature.

 We sincerely appreciate the reviewer’s constructive comments, which helped us improve the mathematical consistency, clarity, and presentation of the manuscript.
